# Heterogeneity in pneumolysin expression governs the fate of *Streptococcus pneumoniae* during blood-brain barrier trafficking

**DOI:** 10.1371/journal.ppat.1007168

**Published:** 2018-07-16

**Authors:** Manalee Vishnu Surve, Smita Bhutda, Akshay Datey, Anjali Anil, Shalini Rawat, Athira Pushpakaran, Dipty Singh, Kwang Sik Kim, Dipshikha Chakravortty, Anirban Banerjee

**Affiliations:** 1 Bacterial Pathogenesis Lab, Dept. of Biosciences and Bioengineering, Indian Institute of Technology Bombay, Powai, Mumbai, INDIA; 2 Dept. of Microbiology and Cell Biology, Indian Institute of Science, Bengaluru, INDIA; 3 Centre for Biosystems Science and Engineering, Indian Institute of Science, Bengaluru, INDIA; 4 National Centre for Preclinical Reproductive and Genetic Toxicology, National Institute for Research in Reproductive Health (ICMR), J. M. Street, Parel, Mumbai, INDIA; 5 Division of Pediatric Infectious Diseases, School of Medicine, Johns Hopkins University, Baltimore, MD, United States of America; St. Jude Children's Research Hospital, UNITED STATES

## Abstract

Outcome of host-pathogen encounter is determined by the complex interplay between protective bacterial and host defense strategies. This complexity further amplifies with the existence of cell-to-cell phenotypic heterogeneity in pathogens which remains largely unexplored. In this study, we illustrated that heterogeneous expression of pneumolysin (Ply), a pore-forming toxin of the meningeal pathogen, *S*. *pneumoniae* (SPN) gives rise to stochastically different bacterial subpopulations with variable fate during passage across blood-brain barrier (BBB). We demonstrate that Ply mediated damage to pneumococcus containing vacuolar (PCV) membrane leads to recruitment of cytosolic “eat-me” signals, galectin-8 and ubiquitin, targeting SPN for autophagic clearance. However, a majority of high Ply producing subset extensively damages autophagosomes leading to pneumococcal escape into cytosol and efficient clearance by host ubiquitination machinery. Interestingly, a low Ply producing subset halts autophagosomal maturation and evades all intracellular defense mechanisms, promoting its prolonged survival and successful transcytosis across BBB, both *in vitro* and *in vivo*. Ply therefore acts as both, sword and shield implying that its smart regulation ensures optimal disease manifestation. Our elucidation of heterogeneity in Ply expression leading to disparate infection outcomes attempts to resolve the dubious role of Ply in pneumococcal pathogenesis.

## Author summary

*Streptococcus pneumoniae*, the Gram-positive diplococci, is the primary etiological agent of bacterial meningitis. In order to cause central nervous system (CNS) infections, SPN has to breach the blood-brain barrier, however, the pneumococcal determinants involved in this process remain unidentified. Here, we demonstrate that pneumolysin, a pore forming toxin secreted by SPN, plays a complex role in governing the fate of the pathogen while trafficking through BBB. Though presumed to be an important virulence attribute, the role of Ply in SPN pathogenesis, particularly in development of meningitis remained debatable. By revealing heterogeneity in Ply expression within isogenic SPN population, our findings attempt to resolve the uncertain role of Ply in SPN pathogenesis. We illustrate that heterogeneity in Ply expression gives rise to stochastically different pneumococcal subpopulations during BBB trafficking. This arises as a consequence of differential interactions of SPN with host microbicidal defense pathways like autophagy and ubiquitination machinery. Among the stochastic SPN population, a low Ply producing SPN subset not only shows improved persistence, but is also capable of successful transcytosis across the BBB resulting in CNS pathogenesis. Overall, our findings suggest that tight spatio-temporal regulation of Ply expression is key to SPN subsistence and dissemination within the host.

## Introduction

The complex battle between the microbial virulence strategies and the host’s counter-defensive mechanisms governs the ultimate outcome of infection. Traditional approaches involving the bulk population based model considers each interacting partner as a functionally homogenous entity, overlooking the stochastic variation in the gene expression between isogenic cells of a population. This stochasticity in virulence attributes results in functionally heterogeneous host and pathogen population subsets which dynamically interact with each other and are responsible for the genesis of different infection foci, triggering disparate infection outcomes [1].

During the course of disease progression, a pathogenic microbe needs to pass through several infection bottlenecks which include initial asymptomatic colonization to further invasion and finally crossing of cellular barriers to infiltrate deeper tissues for establishment of a protective niche. These transitions are accomplished by sensing the ever changing environmental cues and smart regulation of the virulence repertoire by the pathogen that facilitates evasion, manipulation or exploitation of various host immune defense mechanisms witnessed at different stages of infection. In addition to such kind of deterministic adaptation, stochasticity in virulence gene expression within the clonal population gives rise to simultaneous existence of professional subsets that play crucial roles at different stages of host-pathogen interactions, facilitating the establishment of successful infection and disease progression. In this study, we set out to explore the role of heterogeneous expression of a pore-forming toxin, pneumolysin by the meningeal pathogen *Streptococcus pneumoniae* during its transcellular passage through the blood-brain barrier (BBB) [2,3], a crucial step in meningitis pathogenesis.

*Streptococcus pneumoniae* (SPN, pneumococcus), a Gram positive human pathogen, is the common cause of life-threatening invasive diseases such as pneumonia, sepsis and meningitis [4]. In order to cause meningitis, SPN must penetrate the BBB comprised of brain microvascular endothelial cells. Employing a variety of surface anchored proteins SPN attaches and penetrates the BBB [5–10], however, little is known about the mechanistic details behind how SPN thwarts lysosomal killing during transcellular passage across brain endothelium. Following invasion into brain endothelial cells, SPN displays remarkable heterogeneity with respect to the infection outcome. While a majority of the invaded SPN gets killed in a lysosome dependent manner [11], a minor subset of the invaded SPN recycles back to apical surface via recycling endosomes [3]. Yet, another subset of the intracellular SPN is neither killed, nor recycled back, but is transcytosed from the apical to the basolateral side, enabling further dissemination in the host [3]. However, the key virulence genes and their molecular mechanisms underlying these heterogeneous infection outcomes during pneumococcal passage across the BBB remain uninvestigated.

Pneumolysin (Ply), the cholesterol dependent pore-forming toxin secreted by SPN, promotes cell lysis and extensive tissue damage in the host [12]. This multifaceted toxin is involved in a gamete of other cellular activities, such as complement activation, DNA damage, prevention of opsonophagocytosis and induction of apoptosis [13]. However, despite being one of the major virulence factors, its role in pneumococcal pathogenesis remains controversial [14,15]. In this study, we demonstrate that differential expression of pneumolysin gives rise to stochastically different SPN subpopulations inside brain endothelium governing its fate—during transit through the BBB. We demonstrate that among the heterogeneous SPN population that invades the brain endothelium, majority of Ply expressing SPN succumb to intracellular surveillance mechanisms but a handful of low Ply expressing SPN which safely transcytose through the BBB to gain access into the brain, a critical event in pneumococcal disease progression.

## Materials and methods

### Ethics statement

All the experimental work on animals was done as per the guidelines of the Committee for the Purpose of Control and Supervision of Experiments on Animals (CPCSEA), India. The study protocol has been reviewed and approved by the Institutional Animal Ethics Committee (IAEC, Reg. no. 48/1999/CPCSEA) of Indian Institute of Science (IISc, Bengaluru, India) under the project number CAF/Ethics/380/2014. All experimental protocols adhered to the Breeding of and Experiments on Animals (Control and Supervision) Rules, 1998, and its amendments that were published, as a sub section (1, 1A and 2) of Section 17 of the Prevention of Cruelty to Animals Act, 1960 (59 of 1960) under the notification of the Ministry of Environment and Forests, Govt. of India.

### Antibodies and inhibitors

Antibodies used in this study were against; LAMP1 (Cell Signaling Technology, D2D11), Galectin 8 (R & D Systems, AF1305), NDP52 (Abcam, ab68588), Pneumolysin (Santacruz Biotechnology, sc-80500; Abcam, ab71811), GAPDH (Millipore, MAB374), Ubiquitin (Enzo Life Sciences, BML-PW-8810), LC3 (Cell Signaling Technology, 4108S), Phosphorylcholine (Sigma Aldrich, M1421). Antiserum against pneumococcal Enolase was kindly provided by Sven Hammerschmidt (University of Greifswald, Germany). Fine chemicals used in this study include Bafilomycin A1 (B1793), 3-Methyladenine (M9281), Rapamycin (R0395), PYR41 (N2915) and MG132 (474791), from Sigma Aldrich.

### Bacterial strains and constructs

SPN strain R6 (serotype 2, unencapsulated) and TIGR4 (serotype 4, encapsulated) were obtained from Prof. TJ Mitchell (Univ. of Birmingham, UK). Other encapsulated SPN strains, namely, D39 (serotype 2), Tupelo (serotype 14) and A60 (serotype 19F) were generously provided by Prof. EI Tuomanen (St. Jude Childrens Hospital, USA). All SPN strains were routinely grown in Todd-Hewitt broth (THB) supplemented with 1.5% yeast extract (THY media) at 37°C in 5% CO_2_ and when necessary the following antibiotics were used: kanamycin (200 μg/ml), spectinomycin (100 μg/ml), chloramphenicol (2 μg/ml).

To construct the *ply* mutant strain (Δ*ply*), *ply* gene was amplified from the genome of SPN and cloned in the pGEMT Easy vector (Promega). This was further digested with BbsI/AvaI for insertion of a kanamycin resistance cassette. The recombinant vector was linearized with NotI and transformed into wildtype SPN strains (R6 or TIGR4) using competence stimulating peptide 1 or 2 (100 ng/ml). Full length *ply* gene was cloned in between BamHI and SacI sites of the shuttle vector pIB166 [16] (kind gift from Prof. Indranil Biswas, Kansas University Medical Centre, USA) and used for complementation (Δ*ply*:pPly).

High (WT:Ply-High), low (WT:Ply-Low) Ply expressing SPN strains were constructed using the principle as described earlier [17]. Briefly, following cloning of the *ply* gene between BamHI / XhoI sites in pBSK, specific silent point mutations were incorporated in the 5' region of *ply* mRNA by PCR amplification with ply-sdm1-F / ply-sdm1-R and ply-sdm2-F / ply-sdm2-R primer pairs. This manipulated the folding energies of the transcripts affecting their translation efficiencies and resulting in modification of Ply expression. Site directed mutagenesis was also performed to generate W433F variant of Ply, by PCR amplification of Ply ORF cloned in pBSK with ply-F-W433F / ply-R-W433F primers. Individually these *ply* variants were sub-cloned in BamHI/XhoI sites in a recombinant vector where upstream and downstream regions of *ply* gene were sequentially cloned between KpnI / XhoI and BamHI / XbaI sites following amplification with *ply*-up-KpnI-F2 / *ply*-up-XhoI-R2 and *ply*-dwn-BamHI-F2 / *ply*-dwn-XbaI-R primer pairs, respectively, from SPN genome. Finally, a spectinomycin resistance cassette (Sp^r^) was cloned in the BamHI site of the recombinant vectors containing *ply* variant genes coding for either W433F, high or low Ply expression, respectively. The recombinant vectors were linearized with PvuII and used for transformation of WT SPN strain R6 or TIGR4 using either CSP-1 or CSP-2, respectively, to generate high (WT:Ply-High), low (WT:Ply-Low) and W433F (WT:Ply^W433F^) Ply expressing strains.

To transcriptionally fuse *gfp* (green fluorescent protein) with *ply* gene, firstly *ply* gene was PCR amplified using ply-XhoI-F / ply-XmaI-R primer pairs and was cloned into the recombinant vector containing upstream and downstream regions of *ply* gene. Further *gfp* gene was amplified from *hlpA*-GFP fusion cassette (kindly provided by JW Veening, University of Groningen, Netherlands) using GFP-XmaI-F1 / GFP-BamHI-R primers and was cloned in XmaI / BamHI sites downstream of *ply* gene in the recombinant vector. Finally, a Sp^r^ cassette was cloned in the BamHI site of the recombinant vector containing the *ply-gfp* fusion cassette that was used for transformation of WT SPN (R6) following digestion with PvuII and transformants were selected on spectinomycin containing plates.

Capsule was deleted in WT TIGR4 strain using allelic exchange method. Briefly, *dexB* (SP_0342) and *aliA* (SP_0366) gene segments, present upstream and downstream of the capsule locus were sequentially cloned in SacI / BamHI and BamHI / XhoI sites in pBSK following amplification with dexB-F-SacI / dexB-R-BamHI and aliA-F-BamHI / aliA-R-XhoI primer pairs, respectively. A Sp^r^ cassette, was subsequently cloned in the BamHI site of the recombinant vector and the final construct containing Sp^r^ cassette flanked by *dexB* and *aliA* gene segments was used for transformation of WT SPN (TIGR4) and transformants were selected on spectinomycin containing plates.

SPN strains were made fluorescent following transformation with *hlpA*-tRFP and *hlpA*-GFP fusion cassettes (kindly provided by JW Veening, University of Groningen, Netherlands) [18] or by staining with DRAQ5 (BD Biosciences) or DAPI. All gene deletions and cassette insertions were verified by PCR amplification of the respective gene locus followed by DNA sequencing. Unless otherwise mentioned, SPN strain R6 and its derivatives were used for *in vitro* experiments while WT TIGR4 and its derivatives were used for *in vivo* experiments.

### Hemolysis assay

Hemolysis assay was performed by adopting the protocol as described earlier [19]. Briefly, bacterial pellets obtained from mid-exponentially (OD_600nm_ ~ 0.4) grown cultures were lysed by sonication and crude extracts were collected following centrifugation (15000 rpm, 30 min, 4°C). Tenfold dilutions of the bacterial crude extracts (final volume 100 μl) containing equal amount of protein were incubated with 2% RBC suspension in presence of 10 mM DTT. After 60 min incubation at 37°C, 50μl PBS was added to these suspensions and following removal of unlysed RBCs by centrifugation (1500 rpm, 10 min), 100 μl of the supernatants were collected. Absorbance of the released hemoglobin in the supernatants were determined at 540 nm using a microplate spectrophotometer (Thermo Fischer scientific). PBS and Triton X-100 (0.05%) were used as negative and positive control, respectively.

### Flow cytometry

Flow cytometry of SPN cultures was performed as described earlier [20]. Briefly, SPN cultures grown till 0.4 OD_600nm_ were washed with PBS. Approximately 2x10^6^ bacteria were incubated with rabbit anti-Ply antibody (1:50) for 30 min at 4°C. Following washes with PBS, bacteria were incubated with secondary antibody (1:100) for 30 min at 4°C. Finally, bacterial cells were washed with PBS and subjected to flow cytometry using a BD FACS Aria-Fusion flow cytometer using forward and side scatter parameters to gate on atleast 10000 SPN. Results were analysed using FlowJo software (version 9.3) and data was represented as percent of bacterial population positive for fluorescence markers.

### Cell culture and transfections

Human brain microvascular endothelial cells (hBMECs, provided by Prof. KS Kim, Johns Hopkins University) were routinely cultured in RPMI 1640 medium (Gibco) supplemented with 10% Fetal Bovine Serum (Gibco), 10% Nu-Serum (BD Biosciences), and 1% Minimum Essential Medium Non-Essential Amino acids (Gibco) at 37°C in 5% CO_2_ [21]. Stably transfected hBMECs overexpressing GFP-LC3, YFP-Gal8 and mStrawberry-Ubq fusion proteins were constructed by transfecting hBMECs with pBABEpuro GFP-LC3 vector (Addgene, 22405), M6Pblast-YFP-LGALS8 [22] (kindly provided by F Randow, MRC Laboratory of Molecular Biology, UK) and pMRX-IRE-mStrawberry-ubiquitin [23] (kind gift from T Yoshimori, Osaka University, Japan). For RNAi mediated gene knock down, siRNA directed towards galectin 8 (Thermo Fisher Scientific, HSS106038) and scrambled siRNA (Thermo Fisher Scientific, 4390846) were used to transiently transfect hBMECs. All transfections were performed using Lipofectamine 2000 reagent (Thermo Fisher Scientific) and selections were done in the presence of 1 μg/ml puromycin (Sigma Aldrich) and 2 μg/ml blasticidin hydrochloride (HiMedia) where necessary.

### Penicillin gentamicin protection assay

Infection assays were performed as described earlier [7]. Briefly, fully confluent hBMEC monolayers were infected at a multiplicity of infection (MOI) of 1 (for Δ*cps*, Δ*cps* Δ*ply* strains) 10 (for R6 and its derivatives) and 50 (for TIGR4 and its derivatives) for 1 h and further incubated in culture medium containing penicillin (10 μg/ml) and gentamicin (400 μg/ml) for 2 h to kill the extracellular bacteria. After several washes with RPMI, cells were trypsinized (0.025% trypsin EDTA), lysed (0.025% Triton X-100) and serial dilutions of the lysates were plated on BHI (Brain Heart Infusion) agar plates for enumeration of bacterial colonies. Bacterial invasion efficiency was calculated as (recovered CFU/initial inoculum CFU) × 100%. To assess intracellular survival trend of bacteria, cell lysates were prepared similarly and spread plated at indicated time intervals following 1 h of infection and 2 h of antibiotic treatment. Surviving bacteria at different time points were enumerated and was represented as percent survival at indicated time points relative to 0 h (post antibiotic treatment). For inhibition studies, hBMECs were pretreated with Bafilomycin A1 (100 nm, 1 h), 3-Methyladenine (1 mM, 24 h) or PYR41 (45 μM, 1 h) before infection with bacteria.

### MTT assay

hBMEC cells were infected with WT R6 and its Δ*ply* mutant (MOI ~10) for 1 h followed by penicillin-gentamycin treatment as described earlier. At 12 h post infection, cell viability was assessed using MTT assay kit (HiMedia, India) according to manufacturer’s protocol. Uninfected cells were used as negative control while Triton X-100 (0.025%) was used as positive control.

### Bacterial egression assay

hBMEC monolayers were infected with WT:Ply-High and WT:Ply-Low SPN strains (MOI ~ 10) for 1 h followed by penicillin-gentamicin treatment as discussed earlier. After washing the monolayer with RPMI, penicillin (0.04 μg/ml) containing media was added to inhibit bacterial replication. At regular intervals (4 h), the number of bacteria egressed in the culture supernatant were quantified by plating the medium on BHI agar plates. The number of egressed bacteria were expressed as percent egressed at indicated time points relative to number of intracellular bacteria at 0 h (post antibiotic treatment).

### Mouse experiments

The animal experiments were performed by adopting the bacteremia-derived pre-meningitis model as described earlier [24]. Briefly, 6–8 weeks old female Balb/c mice (Jackson Labs) were injected intravenously though tail vein with 200 μl PBS containing 10^6^ bacteria. Control mice were injected with sterile PBS. For survival studies mice were monitored at regular intervals for assessment of disease progression. For analysis of tissue bacterial burden, mice were anaesthetized by intraperitoneal injection of Ketamine and Xylazine at 14 h post infection. Blood was collected by retro-orbital puncture for quantification of pneumococci in the blood. Following blood collection, mice were perfused with sterile PBS in the left ventricle via the vena cava and the blood was allowed to perfuse out from perforation made in the right auricle, until complete flushing out of remaining blood. Brain, lungs and spleen were aseptically collected in PBS. One half of the brain was transferred in 4% paraformaldehyde for histology studies and the other half was further divided into two parts wherein one part was used to prepare homogenates for bacterial enumeration and the other part was transferred into TRIZOL for RNA isolation. Tissue homogenates (prepared by bead beating using 1 mm glass beads) or blood were serially diluted and plated on blood agar for enumeration of bacterial load.

SPN proliferation in the systemic circulation of mice was determined by tail vein blood sampling (10 μl) at selected time points post infection by making an incision with a surgical blade followed by serial dilution and plating on blood-agar for CFU enumeration.

### Fluorescence imaging

For immunofluorescence detection, hBMECs were grown on 1% collagen coated glass cover slips and infected with SPN strains as described above. At desired time points post infection, cells were washed with RPMI, fixed with 4% paraformaldehyde for 15 min followed by permeabilization using 0.1% Triton X-100 for 10 min and blocking in 3% BSA for 2 h, all in PBS at RT. Cells were treated with appropriate primary antibody in 1% BSA in PBS for overnight at 4°C. Next day, cells were washed with PBS and incubated with suitable secondary antibody in 1% BSA in PBS for 1 h at RT. Finally, coverslips were washed with PBS and mounted on glass slides along with VectaShield with or without DAPI (Vector Laboratories) for visualization using a Laser Scanning Confocal microscope (Zeiss Axio-Observer Z1) under 40X or 63X oil objectives. The images were acquired after optical sectioning and then processed using Zen lite software (Version 5.0.). Blood smears of infected mice were prepared on slides and were processed similarly for immunofluorescence detection.

Live cell imaging was performed on spinning disk and laser scanning confocal microscope (Zeiss Axio-Observer Z1). Transfected hBMECs grown on glass bottom petri dishes were infected with SPN as described earlier and time-lapse imaging was performed at multi-position along with optical sectioning under 63x oil immersion objective. Image analysis and processing was done on Zen lite software (Version 5.0.).

For live/dead bacterial staining, tRFP-SPN infected hBMECs were treated with 1 μM Sytox blue for 5 min following permeabilization with 0.1% saponin in 1 mM MOPS/MgCl_2_ buffer for 10 min. Acidotropic dye LysoTracker Deep Red was used at 100 nM for 1 h before imaging to stain lysosomes. Glass bottom petri dishes containing stained and infected hBMECs were visualized under Laser Scanning Confocal microscope (Zeiss Axio-Observer Z1) under 63X oil objectives.

### Transmission electron microscopy

hBMECs were infected with bacteria for 1 h at MOI 50 as described above and following infection cells were washed with 0.1 M sodium cacodylate buffer, fixed with 2% glutaraldehyde (Sigma) and 2% paraformaldehyde in 0.1 M sodium cacodylate buffer (Sigma). Cells were then washed with 0.1 M sodium cacodylate buffer and post fixed with 1% osmium tetroxide in 0.1 M sodium cacodylate buffer (Sigma) for 1 h at 4°C. Following subsequent washes with cacodylate buffer and distilled water, cells were then dehydrated in increasing concentrations of ethanol and propylene oxide and finally embedded in DER 332/732 resin (Electron microscopy services) by polymerization at 70°C. Ultrathin sections (70 nm) were cut using a glass knife on a Leica Microtome (Ultracut R) and stained with 1% uranyl acetate and 0.1% lead citrate and viewed using a transmission electron microscope (Tecnai 12BT, FEI) at 120 KV.

### Western blot analysis

SPN cultures grown to 0.4 OD_600_ were lysed by sonication and crude extracts were collected following centrifugation (15000 rpm, 30 min, 4°C) of the lysates. In case of hBMEC, monolayers (infected or uninfected) were washed several times with PBS and lysed in ice-cold RIPA buffer (50 mM Tris-Cl, pH 7.89, 150 mM NaCl, 1% Triton X-100, 0.5% Sodium deoyxycholate, 1% SDS) containing protease inhibitor cocktail (Sigma Aldirch), Sodium fluoride (10 mM) and EDTA (5 mM). Total cellular proteins were collected following brief sonication and centrifugation of cell lysates. Proteins present in bacterial or hBMEC cell lysates (10 μg or 20 μg) were separated on 8–15% SDS PAGE gels and were then transferred to nitrocellulose membranes (Bio-Rad). Following blocking, incubation with appropriate primary and HRP-conjugated secondary antibodies, blots were developed using ECL substrate (Bio-Rad).

### Histology

Brain tissues obtained from infected or control mice were fixed in 4% paraformaldehyde for 12 h at 4°C. Tissues were then dehydrated and embedded in paraffin. Paraffin-embedded brain tissue were sectioned (5 μm) under a microtome and processed for hematoxylin and eosin (H&E) and Gram staining. Stained sections were analyzed under bright-field microscope (Eclipse Ti, Nikon).

### RNA isolation, cDNA synthesis and quantitative reverse transcriptase PCR

Total RNA from mouse tissue was isolated using TRIzol reagent (ThermoFisher Scientific) as per manufactures protocol. For RNA isolation, tissues were homogenized in a bead beater in 1 ml TRIzol containing 0.1 mm glass beads. RNA was treated for 15 min with DNaseI to remove any DNA contamination and processed for reverse transcription. 5 μg of RNA was reverse-transcribed using SuperScript First-Strand Synthesis System (ThermoFisher Scientific). The cDNA was further used for quantitative RT-PCR (qRT-PCR) for various genes. The sequences of the primers used are mentioned in S1 Table. Changes in the expression of genes relative to 18S rRNA or 16S rRNA (internal controls for mice and SPN, respectively) were analyzed by 2^-ΔΔCt^ method [25].

### Statistical analysis

All statistical analysis were done using GraphPad Prism software (version 5). Statistical tests undertaken for individual experiments are mentioned in the respective figure legends. Statistical significance was accepted at *p* < 0.05.

## Results

### Ply mediated autophagic targeting promotes lower SPN survival and trafficking across BBB

**Fig 1:**
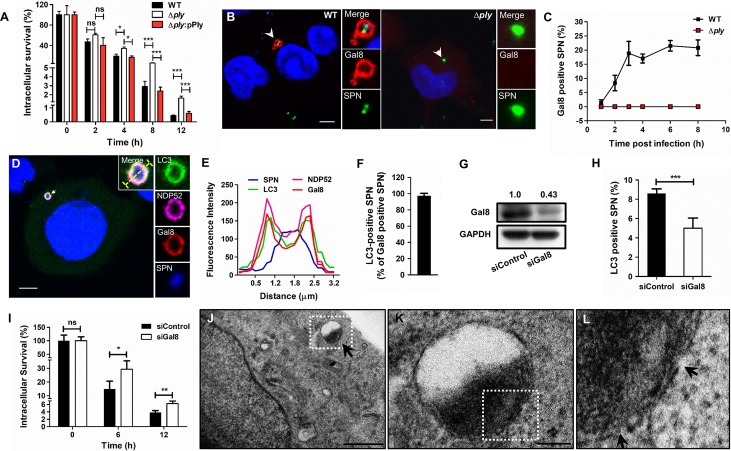
Damage to endosomal membrane by Ply triggers autophagy mediated clearance of SPN in brain endothelium. **A.** Intracellular survival efficiency of WT SPN, Δ*ply* mutant and Δ*ply*:pPly strains of R6 (serotype 2) in hBMECs were calculated as percent survival at indicated time points relative to 0 h. Data are presented as mean ± SD of triplicate experiments. Statistical analysis was performed using one-way ANOVA (Tukey’s multiple comparison test). *p<0.05; ***p<0.001. **B.** Confocal micrographs showing association of Gal8 with GFP expressing WT and Δ*ply* SPN strains at 3 h p.i. in hBMECs. Arrowhead designates bacteria shown in insets. Event localized at z-stack no. 6 and 5 out of 10 and 11 for WT and Δ*ply*, respectively. Scale bar, 5 μm. **C.** Percent co-localization of Gal8 with WT and Δ*ply* SPN strains at indicated time points post-infection. n ≥ 100 bacteria per coverslip. Data are presented as mean ± SD of triplicate hBMEC cultures. **D.** Confocal micrograph showing association of WT SPN (blue) with Gal8 (red), NDP52 (pink) and LC3 (green) at 3 h p.i.. hBMECs stably expressing GFP-LC3 were infected with SPN and stained with anti-Gal8 Ab, anti-NDP52 Ab and DAPI. Arrowhead designates bacteria shown in insets. Event localized at z-stack no. 4 out of 9. Scale bar, 5 μm. **E.** Fluorescent line scan across the yellow line in the merged inset in “**D**”, depicting co-localization of SPN with Gal8, NDP52 and LC3. **F.** Quantification of co-localization of LC3 with Gal8 positive SPN in hBMECs at 3 h p.i. Data are presented as mean ± SD of triplicate experiments. n ≥ 50 bacteria per coverslip. **G.** Western blot demonstrating knock-down in expression of Gal8 in hBMEC cultures following transfection with a targeted siRNA against *LGALS8* (siGal8). GAPDH served as loading control. Relative ratio of Gal8 to GAPDH band intensities (normalized to siControl) is mentioned on top of the blot. **H.** Quantification of LC3 co-localization with WT SPN under Gal8 knockdown conditions using siGal8. Cells transfected with siScrambled served as control (siControl). n ≥ 100 bacteria per coverslip. Data are presented as mean ± SD of triplicate experiments. Statistical analysis was performed using Students t-test. ***p<0.001. **I.** Intracellular survival of WT SPN in hBMECs following transfection with siGal8 or siControl at indicated time points relative to 0 h. Data are presented as mean ± SD of triplicate hBMEC cultures. Statistical analysis was performed using two-way ANOVA (Bonferroni test). ns, non-significant, *p<0.05, **p<0.005. **J–L.** Transmission electron micrograph depicting WT SPN **(J)** in vacuolar compartments inside hBMECs. Scale bar, 1 μm. Zoomed in view of the boxed area in “**J**” and “**K**” are shown in “**K**” (scale bar, 0.2 μm) and “**L**”, respectively. Arrow in “J” points towards SPN sequestered in a vacuole while arrows in “L” show disruptions in the PCV membrane.

To explore the direct role of Ply in pneumococcal intracellular lifestyle during trafficking through the BBB, we firstly performed an intracellular survival assay with the SPN WT and *ply* mutant (Δ*ply*) strains, which was developed in serotype 2 strain R6 via allelic exchange that lacked hemolysis activity (S1A Fig). The Δ*ply* mutant strain exhibited higher survival ability particularly at later time points of 8 and 12 h, wherein it showed 2 and 3.5 fold higher survival efficiency compared to WT SPN (Fig 1A) without any observable cell death due to infection (S1B Fig). This higher survival phenotype of Δ*ply* mutant was completely reversed back to that of the WT by ectopic expression of Ply from a complementing plasmid in Δ*ply* mutant strain (Fig 1A). Similar phenotype was observed in case of both Δ*ply* and Δ*cps*Δ*ply* double mutant in comparison to encapsulated WT serotype 4 strain TIGR4 and its capsule mutant strain, Δ*cps*, respectively (S1C–S1E Fig), suggesting a capsule/serotype-independent detrimental role of pneumolysin in intracellular survival of SPN during BBB trafficking.

Next, we used a bacteremia-derived meningitis mouse model to study the role of Ply in BBB crossover [24]. Comparison of bacterial counts in different tissue homogenates and blood at 14 h p.i., revealed that SPN TIGR4 Δ*ply* strain showed lower bacterial counts in all tested tissues as well as in the blood compared to WT TIGR4 (S2A Fig), an observation which corroborated with earlier findings [10]. However, only the brain/blood ratio for Δ*ply* mutant was higher with respect to WT (S2B Fig) compared to other tissues (S2C & S2D Fig). Higher brain/blood ratio in spite of lower pneumococcal burden in respective tissues highlights efficient BBB trafficking potential of Δ*ply* mutant compared to WT SPN strain. Examination of pro-inflammatory response by qRT-PCR revealed no significant difference in the transcript levels of pro-inflammatory cytokines such as, TNFα, IL-1β and IFNγ in the brain tissues of mice that were infected with either WT or Δ*ply* strain (S2E Fig). We also did not observe the typical meningitis hallmarks, such as characteristic leukocyte infiltration in the brain tissue of mice infected with either WT or Δ*ply* strains (S2F Fig). Thus, these observations for both histology and inflammatory response studies support our pre-meningitis animal model, which is characterized by an intact and non-leaky BBB, a prerequisite for investigating the role of Ply in pneumococcal BBB trafficking exclusively via transcytosis route.

We next hypothesized that Ply, by virtue of its pore-forming ability, might puncture pneumococcus containing vacuoles (PCVs) leading to recruitment of cytosolic “eat-me” signal Galectin 8 (Gal8) which binds to exposed glycans lining the luminal surface of bacteria containing vacuoles. This targets the bacteria residing inside the compromised vacuole towards anti-bacterial autophagy [22]. Therefore, we analyzed co-localization of WT and Δ*ply* SPN strains following internalization in hBMECs with Gal8 using confocal microscopy. Clear association of Gal8 with WT SPN was observed while Δ*ply* infected cells failed to show any such events (Fig 1B). This association steadily increased from 1 h to 3 h p.i. and subsequently remained fairly constant (Fig 1C). Moreover, the presence of capsule did not affect the outcome of association of encapsulated serotype 4 SPN strain TIGR4 or its Δ*ply* mutant with Gal8 (S1F Fig). TEM analysis also displayed intermittent rupture in the vacuolar membrane enclosing most of the WT SPN (Fig 1J–1L), further substantiating the role of Ply in loss of PCV membrane integrity. The role of pore-forming activity of Ply in loss of endosomal membrane integrity was also examined using a Ply^W433F^ mutant, which though expresses Ply at similar levels to that of WT, but have completely lost the hemolytic activity (S3A & S3B Fig). Surprisingly, SPN strain containing Ply^W433F^ exhibited equal amount of Gal8 association compared to that of WT (S3C Fig), suggesting that hemolytic activity can’t always be synonymously used for pore formation. Additionally, we observed no difference in intracellular persistence ability of WT:Ply^W433F^ compared to WT SPN (S3D Fig). Visualization of hBMECs infected with WT SPN under a confocal microscope revealed recruitment of both the autophagy receptor NDP52 and the classical autophagosomal marker LC3B on Gal8 marked PCVs (Fig 1D & 1E). Approximately 97% of the Gal8 positive PCVs acquired GFP-LC3B at 3 h p.i. (Fig 1F) indicating faithful autophagic targeting of SPN residing within damaged vacuoles. Pneumolysin seemed to be the sole virulence factor responsible for autophagic targeting of SPN as association with GFP-LC3B is completely abolished in Δ*ply* mutant strain while WT SPN progressively recruited GFP-LC3B until 6 h p.i. which persisted till 10 h p.i. (S4A & S4B Fig). However, we did not observe any significant difference in LC3 flux (LC3I to LC3II conversion) between WT and Δ*ply* strains at both 3 and 6 h p.i. (S4D Fig).

We further examined for the contribution of Ply induced autophagy in higher killing of WT SPN inside hBMECs. Intracellular survival of WT SPN in hBMECs pretreated with the autophagy inhibitor, 3-methyladenine, was found to be 3.8 and 2.6 fold higher compared to untreated cells at 6 and 12 h time points, respectively (S4C Fig). Additionally, siRNA-mediated specific knock down of Gal8 in hBMECs (knock-down efficiency ~60%) (Fig 1G) resulted in significant decrease of LC3 association with WT SPN at 3 h p.i. (Fig 1H) and considerably improved intracellular recovery of the same following infection at both 6 and 12 h time points (Fig 1I). Expectedly, we did not observe any perturbation in the intracellular persistence ability of the Δ*ply* mutant strain following Gal8 knock-down by siRNA (S5A Fig).

Collectively, these findings demonstrate that Ply indeed damages PCV membrane integrity inside brain endothelium which triggers recruitment of the autophagy eat-me signal Gal8 and drives intracellular SPN towards autophagic killing which seems to be detrimental for its trafficking across BBB.

### Variable maturation kinetics of SPN containing autophagosomes

**Fig 2:**
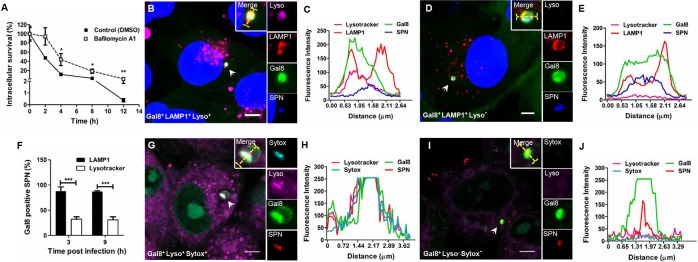
Lysosomal targeting of SPN sequestered autophagosomes during BBB trafficking. **A.** Percent intracellular survival of internalized WT SPN in presence of lysosomal inhibitor Bafilomycin A1 (100 nM) at indicated time points relative to 0 h. Data are presented as mean ± SD of triplicate hBMEC cultures. Statistical analysis was performed using two-way ANOVA (Bonferroni test). *p<0.05, **p<0.005. **B & D**. Confocal micrograph showing association of Gal8 (green) positive WT SPN (blue) with LAMP-1 (red) and LysoTracker (pink) at 3 h p.i. hBMECs stably expressing YFP-Gal8 were stained with Anti-LAMP1 Ab, acidotropic dye LysoTracker Deep Red and DAPI. Arrowhead designates bacteria shown in insets. Event localized at z-stack no. 4 (out of 9) and 6 (out of 10) for “**B**” and “**D**”. Scale bar, 5 μm. **C & E.** Fluorescent line scan across the yellow line in the merged inset in “**B**” and “**D**” are shown in “**C**” and “**E**”, respectively. **F.** Quantification of LAMP1 or LysoTracker association with Gal8 positive SPN in hBMECs at 3 and 9 h p.i. n ≥ 50 bacteria per coverslip. Data are presented as mean ± SD of triplicate experiments. Statistical analysis was performed using two-way ANOVA (Bonferroni test). ***p<0.001. **G & I.** Confocal micrograph showing association of WT SPN (red) with Gal8 (green), LysoTracker (pink) and Sytox (cyan) at 6 h p.i. hBMECs stably expressing YFP-Gal8 were infected with WT SPN constitutively expressing tRFP (red) and stained with LysoTracker (100 nM) and Sytox (1 μM). Arrowhead designates bacteria shown in insets. Event localized at z-stack no. 7 (out of 9) and 6 (out of 8) for “**G**” and “**I**”. Scale bar, 5 μm. **H & J.** Fluorescent line scan across the yellow line in the merged inset in “**G**” and “**I**” are shown in “**H**” and “**J**”, respectively.

It is evident from the co-localization kinetics of SPN with Gal8 and LC3 that Ply induced marking of intracellular SPN for autophagic degradation begins as early as 2 h p.i. Our confocal microscopy analysis also revealed that approximately 63% of WT SPN co-localized with late endosomal marker, lysosome-associated membrane protein 1 (LAMP-1), by 1 h p.i. as compared to only 21% in case of Δ*ply* mutant strain (S5B Fig). Moreover, pretreatment of hBMECs with Bafilomycin A1 (100 nM), a vATPase inhibitor used to halt vacuole acidification, resulted in significant increase in intracellular survival of WT SPN (3 fold at 4 h and 5.5 fold at 8 and 12 h) compared to the non-treated control (Fig 2A). These suggest the remarkable contribution of Ply in driving PCV maturation and targeting the SPN towards lysosomal degradation soon after invasion inside the brain endothelium.

However, despite this, few WT SPN manage to survive up until 12 h and persistently co-localize with autophagosomes (Gal8^+^ and LC3^+^ compartments) till extended hours post infection (8 and 10 h p.i.) (Fig 1C & S4B Fig). To shed light on these paradoxical observations, we explored the maturation of SPN containing autophagosomes by evaluating the interaction of Gal8 positive SPN with LAMP-1 and LysoTracker, an acidotropic probe. Interestingly, we observed that WT SPN was confined within both acidic (Lyso^+^) as well as non-acidic (Lyso^-^) Gal8^+^ LAMP-1^+^ compartments (Fig 2B–2E). At 3 h p.i., approximately 87% of Gal8 positive SPN was associated with LAMP-1 while only 32% of them showed LysoTracker accumulation (Fig 2F). However, the persistence of similar statistics even at 9 h p.i., implies that although Ply mediated pore-formation on PCVs targets few SPN towards Gal8 mediated autophagic degradation indicated by Sytox Blue staining, a dead cell stain (Gal8^+^ Lyso^+^ Sytox^+^), few SPN simultaneously persist inside non-degradative autophagosomes (Gal8^+^ Lyso^**−**^Sytox^**–**^) (Fig 2G–2J), supporting their prolonged survival inside hBMECs observed earlier.

**Fig 3:**
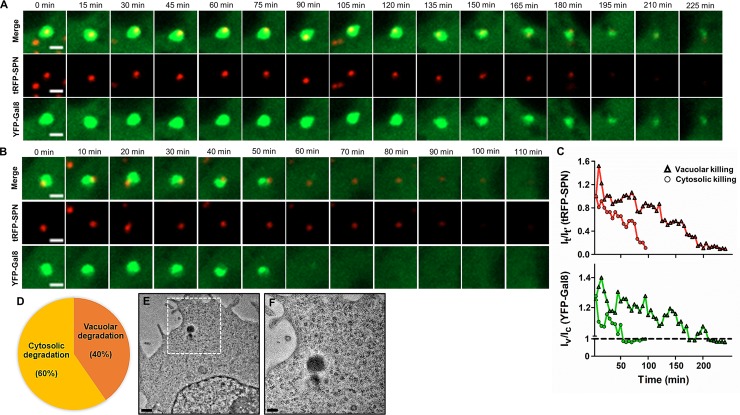
Delay in SPN containing autophagosomal maturation and escape into cytosol. **A & B.** Representative time-lapse montage for degradation of SPN inside Gal8 marked autophagosomes **(A)** or in cytosol **(B)**. hBMECs stably expressing YFP-Gal8 (green) were infected with WT SPN constitutively expressing tRFP (red) and live imaging of infected cells was performed for extended hours beginning at 3 h p.i. under a confocal microscope. Time-lapse series with intervals of 15 min **(A)** or 10 min **(B)** are represented. The stills in “**A**” and “**B**” correspond to movies shown as S1 and S2 Movies, respectively. Scale bar, 2 μm. **C.** Comparison of temporal quantification of fluorescence intensities of Gal8 (lower graph) and SPN (upper graph) in “**A**” and “**B**” relative to either cytosol (I_v_/I_c_ for Gal8, where I_v_ and I_c_ are fluorescence intensities in vacuole and cytosol, respectively) or SPN fluorescence at 0 min (I_t_/I_t'_ for SPN, where I_t_ and I_t'_ are fluorescence intensities of the Gal8 marked SPN at a given time point or 0 min, respectively). **D.** Percentage of intracellular SPN undergone vacuolar or cytosolic degradation as examined by time-lapse imaging of hBMECs stably expressing YFP-Gal8 following infection with tRFP expressing SPN. n = 57. **E & F.** Transmission electron micrograph depicting presence of WT SPN in cytosol without any membrane limiting structures **(E)**. Scale bar, 0.5 μm. Zoomed in view of the boxed area in “**E**” is shown in “**F**”. Scale bar, 0.2 μm.

Further, to gain deeper insights into the maturation kinetics of SPN inside autophagosomes, we monitored tRFP expressing WT SPN in YFP-Gal8 marked compartments in real time using time-lapse confocal microscopy from 3 h p.i. for extended hours. Surprisingly, we observed two different phenomena with respect to the fate of the SPN inside YFP-Gal8 compartments. In one of the phenomena, both tRFP signal of SPN and YFP signal of Gal8 positive compartments faded very slowly with time (Fig 3A and S1 Movie), implying gradual degradation of SPN inside autophagosomes. While in the other phenomenon, early fading and collapse of Gal8 signal was accompanied with persistent SPN signal for longer duration, suggesting disruption of Gal8 positive compartments and escape of SPN into cytosol (Fig 3B and S2 Movie). However, the eventual fading of SPN signal in the cytosol indicates its degradation following recognition and action by other host cytosolic defense mechanisms (Fig 3C). Quantification of such events revealed that approximately 60% of SPN escaped from autophagosomes and subsequently underwent cytosolic degradation, while rest (~ 40%) succumbed to the autophagic trap (Fig 3D). Collectively these findings suggest that following internalization in brain endothelium, despite Ply mediated autophagic targeting of SPN, few pneumococci halt autophagosomal maturation before eventual degradation, while others manage to escape autophagy by translocating into the cytosol.

### Ply induced autophagosomal membrane damage leads to cytosolic escape and ubiquitination-mediated killing of SPN

**Fig 4:**
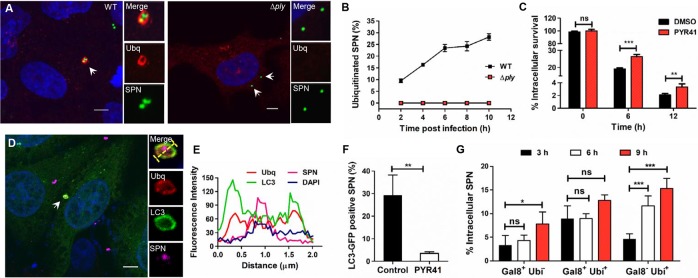
Ply dependent ubiquitination of SPN triggers autophagic clearance. **A.** Confocal micrographs showing association of ubiquitin (red) with GFP expressing WT and Δ*ply* SPN strains (green) inside hBMECs. Arrowhead designates bacteria shown in insets. Event localized at z-stack no. 7 (out of 16) and 7 (out of 12) for WT and Δ*ply*, respectively. Scale bar, 5 μm. **B.** Quantification of association of ubiquitin with WT and Δ*ply* SPN strains at indicated time points p.i. in hBMECs. n ≥ 100 bacteria per coverslip. Data are presented as mean ± SD of triplicate hBMEC cultures. **C.** Intracellular survival of WT SPN in hBMECs following pretreatment with E1 ubiquitin activating enzyme inhibitor PYR41 (45 μM) at indicated time points relative to 0 h. Data are presented as mean ± SD of triplicate hBMEC cultures. Statistical analysis was performed using two-way ANOVA (Bonferroni test). ns, non-significant, **p<0.005, ***p<0.001. **D.** Confocal micrograph showing association of WT SPN (pink) with ubiquitin (red) and LC3 (green) at 3 h p.i. hBMECs stably expressing GFP-LC3 were infected with SPN and stained with anti-Enolase Ab, anti-Ubq Ab and DAPI. Arrowhead designates bacteria shown in insets. Event localized at z-stack no. 7 (out of 16). Scale bar, 5 μm. **E.** Fluorescent line scan across the yellow line in the merged inset in “**D**”, depicting co-localization of SPN with Ubq and LC3. **F.** Quantification of association of LC3 with WT SPN at 6 h p.i. following inhibition of ubiquitination by pretreatment of hBMECs with PYR41 (45 μM). n ≥ 100 bacteria per coverslip. Data are presented as mean ± SD of triplicate experiments. Statistical analysis was performed using Students t-test. **p<0.005. **G.** Quantification of association of Gal8 and ubiquitin with intracellular SPN at 3, 6 and 9 h p.i. Three different population of SPN could be detected, Gal8^+^ Ubq^**–**^, Gal8^+^ Ubq^+^ and Gal8^**–**^ Ubq^+^. n ≥ 100 bacteria per coverslip. Data are presented as mean ± SD of triplicate hBMEC cultures. Statistical analysis was performed using two-way ANOVA (Bonferroni test). ns, non-significant, *p<0.05, ***p<0.001.

Given that SPN escapes from autophagosomes, we speculated that Ply mediated extensive membrane damage might rupture PCVs leading to cytosolic invasion of SPN. Our transmission electron microscopic analysis displaying presence of WT SPN without any membrane limiting structures in the cytosol of hBMECs supported this hypothesis (Fig 3E & 3F). On the contrary, Δ*ply* mutant strain was always observed sequestered in membrane bound vacuolated structures (S5C & S5D Fig). The fact that Ply disrupts autophagosomes leading to escape of SPN into cytosol, propelled us to investigate the possible role of host ubiquitination machinery in combating SPN infection inside brain endothelium. Confocal microscopy showed clear co-localization of ubiquitin (Ubq) with WT SPN inside hBMECs while Δ*ply* mutant strain failed to show any such events, indicating the critical role of Ply in ubiquitination of SPN (Fig 4A). This association between Ubq and WT SPN increased progressively up until 6 h p.i. and persisted for extended hours (Fig 4B). Additionally, most of the ubiquitin positive SPN showed association with LC3-GFP (Fig 4D & 4E). Inhibition of ubiquitination by pretreatment of hBMECs with PYR41, an E1 ubiquitin activating enzyme inhibitor, not only improved the intracellular survival of WT SPN (Fig 4C), but also significantly lowered its association with LC3 (Fig 4F), further confirming the contribution of ubiquitin as another eat-me signal, in addition to galectin-8, targeting SPN towards autophagic degradation. Expectedly, PYR41 inhibition of cellular ubiquitination did not have any effect on the intracellular recovery of Δ*ply* mutant (S5E Fig), justifying our findings that Ply mediated damage is responsible for ubiquitination and subsequent degradation of intracellular SPN. Next, in order to determine the existence of any intersection between these two eat-me signals, we quantified SPN co-localization events with Gal8 and ubiquitin at different time points post infection. Confocal microscopic analysis revealed that as compared to Gal8^+^ Ubq^**−**^and Gal8^+^ Ubq^+^ SPN population, Gal8^**–**^ Ubq^+^ population, which unequivocally represents free cytosolic bacteria that escaped from disrupted vacuoles inside hBMECs, significantly increased from 3 h p.i. to 9 h p.i. (Fig 4G).

**Fig 5:**
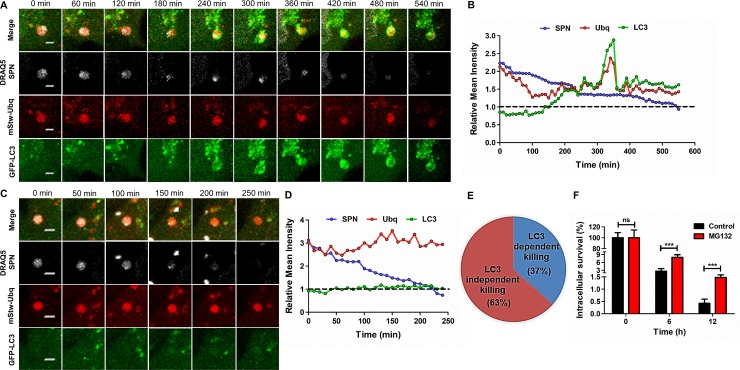
Degradation of cytosolic SPN via autophagy–dependent or independent pathway is reliant on Ply induced ubiquitination. **A, C.** Representative time-lapse montage for degradation of cytosolic SPN following escape from autophagosomes in LC3-dependent **(A)** or independent manner **(C)**. hBMECs stably expressing LC3-GFP (green) and Ubq-mStrawberry (red) were infected with DRAQ5 stained WT SPN (white). Live imaging of infected cells was performed for extended hours beginning at 6 h p.i. under a confocal microscope and time-lapse series with intervals of 60 min **(A)** or 50 min **(C)** are represented. The stills in “**A**” and “**C**” correspond to movies shown as S3 and S4 Movies, respectively. Scale bar, 2 μm. **B, D.** Temporal quantification of ubiquitin, LC3 and SPN fluorescence intensities either relative to fluorescence in the cytosol (Cyt) (for LC3 and Ubq) or fluorescence signal of hBMEC nuclei (Nuc) (for SPN). Relative mean intensity for LC3 (I_PCV_/I_Cyt_) in case of ubiquitinated SPN displayed values > 1.0, depicting LC3-dependent killing of SPN **(B)**. Ubiquitinated SPN that is degraded in LC3 independent manner, displayed relative mean intensity value ~ 1.0 for LC3 **(D)**. In both cases relative mean intensity values for bacteria (I_SPN_/I_Nuc_) remains > 1.0 for substantially long period of time before finally disappearing. **E.** Percentage of ubiquitinated SPN undergone LC3-dependent or independent killing as examined by time-lapse imaging of hBMECs stably expressing GFP-LC3 and Ubq-mStrawberry following infection with DRAQ5 stained SPN. n = 41. **F.** Intracellular survival percentages of WT SPN following treatment with MG132 (10 μM) at indicated time points relative to 0 h. Data are presented as mean ± SD of triplicate experiments. Statistical analysis was performed using two-way ANOVA (Bonferroni test). ns, non-significant, ***p<0.001.

To further determine the mechanism behind ubiquitin-mediated degradation of cytosolic SPN, time-lapse live-cell imaging was performed on hBMECs stably expressing LC3-GFP and Ubq-mStrawberry following infection with DRAQ5 stained WT SPN. Ubq–mStrawberry positive SPN (negative for LC3-GFP), representing cytosolic bacteria, were tracked at 6 h p.i. and monitored for extended hours. Approximately 37% of ubiquitinated SPN displayed accumulation of LC3-GFP with time and eventually degraded while remaining associated with LC3-GFP, representing the contribution of ubiquitin-mediated autophagy in clearance of cytosolic SPN (Fig 5A, 5B & 5E and S3 Movie). Surprisingly, we also observed that 63% of ubiquitinated SPN failed to recruit LC3-GFP and degraded while remaining associated with Ubq-mStrawberry (Fig 5C, 5D & 5E and S4 Movie). Further, treatment with MG132, a 26S proteasomal subunit inhibitor, significantly improved intracellular survival of WT SPN (Fig 5F) suggesting involvement of proteasome-ubiquitin system in promoting SPN clearance via an ubiquitin mediated autophagy-independent pathway. Collectively, these results indicate that Ply-mediated progressive damage on PCV membrane leads to recruitment of eat-me signals like galectin-8 and ubiquitin. Further damage triggers collapse of the autophagosomes and escape of SPN in cytosol where ubiquitination machinery targets them towards autophagy-dependent or autophagy-independent degradative pathways.

### Heterogeneity in Ply expression gives rise to stochastic pneumococcal population inside BBB with variable fate

**Fig 6:**
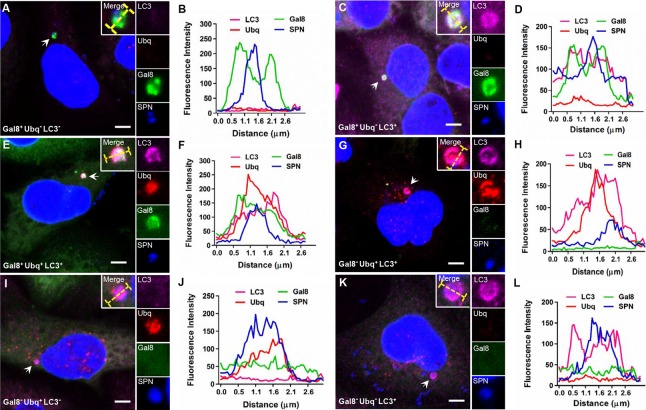
Phenotypic heterogeneity of SPN inside brain endothelium. Confocal images of different population of WT SPN inside hBMECs at 6 h p.i. **(A)** Gal8^+^ Ubq^**–**^LC3^**–**^, **(C)** Gal8^+^ Ubq^**−**^LC3^+^, **(E)** Gal8^+^ Ubq^+^ LC3^+^, **(G)** Gal8^**–**^ Ubq^+^ LC3^+^, **(I)** Gal8^**–**^ Ubq^+^ LC3^**–**^, **(K)** Gal8^**–**^ Ubq^**−**^LC3^+^. hBMECs stably expressing Gal8-YFP were infected with WT SPN (blue) and stained with anti-LC3 Ab (pink) and anti-Ubq Ab (red). Fluorescent line scans across the yellow lines in the merged insets in “**A, C, E, G, I** and **K**”, depicting association of SPN with Gal8, Ubq and LC3 are given in “**B, D, F, H, J** and **L**”, respectively.

Our results clearly demonstrated the existence of phenotypically heterogeneous SPN subsets during trafficking through BBB with regards to variable maturation inside autophagosomes and distinct localization inside brain endothelium (vacuolated or cytosolic) culminating in differential survival ability. To support this hypothesis, we analyzed for the existence of different sub-populations of WT SPN for their association with Gal8, Ubq and LC3 markers inside hBMECs using confocal microscopy. Interestingly, we observed six different sub-populations of SPN inside hBMECs: Gal8^+^ Ubq^**−**^LC3^-^, Gal8^+^ Ubq^**−**^LC3^+^, Gal8^+^ Ubq^+^ LC3^+^, Gal8^**–**^ Ubq^+^ LC3^+^, Gal8^**–**^ Ubq^+^ LC3^**–**^ and Gal8^**–**^ Ubq^**−**^LC3^+^ (Fig 6 and S6A Fig).

**Fig 7:**
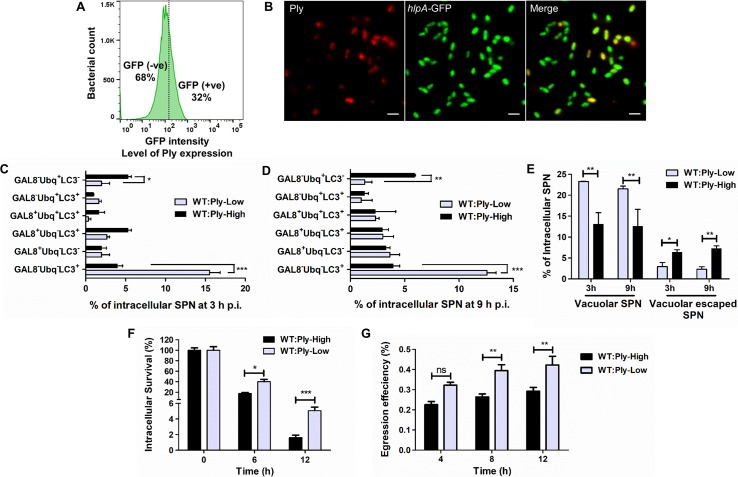
Differential Ply expression governs the spatio-temporal location, survival and trafficking of SPN across BBB. **A.** Flow cytometry of a recombinant SPN strain with *ply* gene transcriptionally fused with *gfp* to monitor variation in Ply expression among isogenic SPN population. **B.** Confocal micrographs depicting heterogeneous Ply expression within a population of *in vitro* grown SPN culture. GFP expressing SPN (*hlpA*-GFP integrated in genome) was probed with Anti-Ply Ab. Scale bar, 1 μm. **C—D.** Quantification of different population subsets of intracellular WT:Ply-High and WT:Ply-Low SPN strains at 3 h **(C)** and 9 h **(D)** p.i. for association with Gal8, Ubq and LC3. n ≥ 100 bacteria per coverslip. Data are presented as mean ± SD of triplicate experiments. Statistical analysis was performed using two-way ANOVA (Bonferroni test). *p<0.05, **p<0.005, ***p<0.001. **E.** Intracellular location of WT:Ply-High and WT:Ply-Low SPN strains at 3 and 9 h p.i. in hBMECs. Gal8^+^ Ubq^**−**^LC3^-^, Gal8^+^ Ubq^**−**^LC3^+^, Gal8^+^ Ubq^+^ LC3^+^ and Gal8^**–**^ Ubq^**−**^LC3^+^ constituted vacuolar SPN population, while Gal8^**–**^ Ubq^+^ LC3^+^ and Gal8^**–**^ Ubq^+^ LC3^**–**^ constituted vacuolar escaped SPN population. n ≥ 100 bacteria per coverslip. Data are presented as mean ± SD of triplicate hBMEC cultures. Statistical analysis was performed using two-way ANOVA (Bonferroni test). *p<0.05, **p< 0.005. **F.** Comparison of intracellular survival efficiencies between WT:Ply-High and WT:Ply-Low strains in hBMECs expressed as percent survival at indicated time points relative to 0 h. Data are presented as mean ± SD of triplicate experiments. Statistical analysis was performed using two-way ANOVA (Bonferroni test); *p <0.05, ***p <0.001. **G.** Comparison of egression efficiencies of WT:Ply-High and WT:Ply-Low SPN strains across confluent hBMEC monolayers at indicated time points. Data are presented as mean ± SD of triplicate experiments. Statistical analysis was performed using two-way ANOVA (Bonferroni test); ns, non-significant, **p< 0.005.

This prompted us to speculate that existence of heterogeneity in Ply expression among isogenic SPN population and the resulting variability in the extent of Ply mediated damage on PCV membrane, may give rise to all such spatio-temporally heterogeneous infection outcomes. Flow cytometric analysis with a SPN strain expressing the fluorescent reporter GFP, transcriptionally fused with the *ply* gene revealed a normal distribution curve for GFP fluorescence, representing wide variation in Ply expression from one cell to another (Fig 7A). This recombinant SPN strain showed similar hemolytic ability as that of WT SPN (S6B Fig). Moreover, the surface Ply expression assessed by anti-Ply antibody in this transcriptional fusion strain also exhibited heterogeneity by flow cytometry (S6C Fig). Similar variance in Ply expression was observed when *in vitro* grown WT SPN culture was stained with anti-Ply antibody and checked with fluorescence microcopy (Fig 7B), supporting the existence of low to high Ply producers within isogenic SPN population. Propelled by this finding of heterogeneity in Ply expression, we constructed two SPN strains (WT:Ply-High and WT:Ply-Low) by introducing specific point mutations in the 5' region of *ply* mRNA that manipulated the folding energies of the transcripts [17], affecting their translation efficiencies and resulting in differential Ply expression, both intracellularly as well as on cell surface (S7A–S7D Fig). We then quantified the intracellular SPN population subsets observed earlier (Fig 6) in hBMECs following infection with WT:Ply-High and WT:Ply-Low strains at 3 h p.i. and 9 hp.i. (Fig 7C & 7D). Classification of these subsets with regards to cellular location of SPN showed that vacuolated SPN population (includes Gal8^+^ Ubq^**–**^LC3^**–**^, Gal8^+^ Ubq^**−**^LC3^+^, Gal8^+^ Ubq^+^ LC3^+^ and Gal8^**–**^ Ubq^**−**^LC3^+^) is significantly higher in case of WT:Ply-Low strain while vacuolar escaped SPN population (includes Gal8^**–**^ Ubq^+^ LC3^+^ and Gal8^**–**^ Ubq^+^ LC3^**–**^) is substantially higher in case of WT:Ply-High strain (Fig 7E). Out of these subsets; Gal8^**–**^ Ubq^+^ LC3^**–**^ and Gal8^**–**^ Ubq^**−**^LC3^+^ showed significant difference between the two strains as compared to all other subsets (Fig 7C & 7D). The Gal8^**–**^ Ubq^+^ LC3^**–**^ which unequivocally represents the cytosolic SPN pool, was significantly higher in hBMECs infected with WT:Ply-High strain. Contrarily, substantially higher occurrence of the Gal8^**–**^ Ubq^**−**^LC3^+^ subset was observed in case of WT:Ply-Low strain. This unique subset was devoid of association with any of the autophagy eat-me signals and predominated at both the time points (3 h p.i. and 9 h p.i.) compared to all other subsets. We then compared the intracellular survival efficiencies of these two SPN strains. Significantly higher survival ability of the WT:Ply-Low strain was observed compared to WT:Ply-High strain at 12 h (3.1 fold) time point (Fig 7F), though they exhibited similar invasion efficiencies (S7E Fig). Furthermore, WT:Ply-Low SPN strain egressed out of hBMECs in significantly larger numbers as compared to WT:Ply-High SPN strain, suggesting higher transcytosis efficiency of low Ply producing SPN (Fig 7G).

Collectively, these results suggest that the low Ply producing subset of SPN preferentially stays compartmentalized by minimizing damage on SPN containing autophagosomes (predominated by Gal8^**–**^ Ubq^**−**^LC3^+^ subset) and shows prolonged survival and trafficking across the brain endothelium.

### Low Ply expression promotes safe passage of SPN across brain endothelium *in vivo*

**Fig 8:**
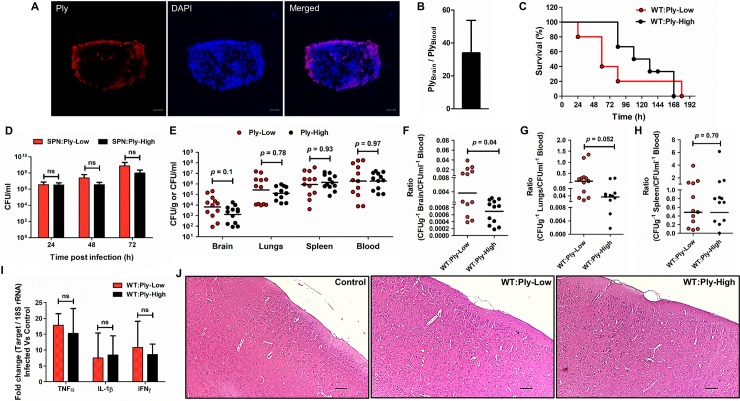
Low Ply expression promotes pneumococcal trafficking across BBB. Balb/c mice were infected via i.v. route with 10^6^ CFU of WT, WT:Ply-High or WT:Ply-Low SPN strain and at 14 h p.i. animals were sacrificed. PBS injected mice served as control. **A.** Confocal micrographs depicting heterogeneous Ply expression (red) within WT SPN population (blue) in the blood of infected mice. Blood smears were stained with Anti-Ply antibody and DAPI. Scale bar, 5 μm. **B.** Ratio of transcript levels of Ply in brain to blood of mice infected with WT SPN. Transcript levels were normalized to 16S rRNA and expressed as fold change compared to Ply transcript levels of WT SPN grown *in vitro* in THY medium. **C.** Kaplan-Meier survival curve of mice (n = 5 per group) following i.v. injection with different SPN strains. Experiments were repeated twice and one representative graph is shown. Statistical analysis was performed using Log-rank test. **D.** Net growth of SPN strains in the blood of infected mice (n = 5 per group) at indicated time points post infection. Experiments were repeated twice and one representative graph is shown. Statistical analysis was performed using two-way ANOVA (Bonferroni test); ns, non-significant. **E.** Quantification of bacterial counts (CFU) in various tissue homogenates and blood of mice infected with different Ply expressing SPN strains. Each dot represents one mouse; black bars show average values. n = 12 per group (2 experiments with n = 6 per group). Statistical analysis for each tissue was individually performed using non-parametric test (Mann-Whitney test). *p* values are mentioned in the graph. **F—H.** Ratio of bacterial CFU in brain to blood **(F)**, lungs to blood **(G)** and spleen to blood **(H)** of individual infected mice. Each dot represents one mouse; black bars show average values. n = 12 per group (2 experiments with n = 6 per group). Statistical analysis was performed using non-parametric test (Mann-Whitney test). *p* values are mentioned in the graph. **I.** Transcript abundance of pro-inflammatory cytokines in total RNA isolated from brains of mice that were injected with PBS or infected with different SPN strains. Transcript levels were normalized to 18S rRNA and expressed as fold change compared to control mice. Statistical analysis was performed using two-way ANOVA (Bonferroni test); ns, nonsignificant. **J.** Histopathology of H & E stained representative brain tissue samples of control (PBS injected) or mice infected with different strains of SPN. Scale bar, 100 μm.

We next investigated the role of heterogeneous Ply expression in BBB trafficking of SPN using the previously described bacteremia-derived meningitis mouse model of infection. Following infection via hematogenous route, WT SPN in the blood displayed enormous heterogeneity in Ply expression, wherein majority of them showed low to negligible Ply levels at 14 h p.i. (Fig 8A). Examination of Ply transcript levels in the brain and blood of WT SPN infected mice further supported the abundance of low Ply expressing SPN in the blood (Fig 8B). We next compared the contribution of WT:Ply-High and WT:Ply-Low strains (in TIGR4 background) to SPN CNS pathogenesis. Survival studies revealed significant early death of mice infected with WT:Ply-Low strain. The median survival time of the WT:Ply-Low infected mice (60 h) was two-fold lower compared with WT:Ply-High infected mice (120 h) (Fig 8C). We also analyzed proliferation of different SPN strains in the mice blood at early time points during the course of infection. Insignificant difference in blood bacterial load between WT:Ply-Low and WT:Ply-High strains at all time points tested ruled out the possibility of sepsis induced early death of mice infected with WT:Ply-Low strain (Fig 8D). We then compared the capability of WT:Ply-High and WT:Ply-Low strains to disseminate in various mice tissues. Assessment of bacterial load in mice tissues revealed higher SPN counts only in brain (6.69x10^3^ vs 1.29x10^3^) of animals that were infected with WT:Ply-Low strain, however no such difference was observed in case of and lungs (2.71x10^5^ vs 1.78x10^5^), spleen (8.9x10^5^ vs 14.1x10^5^) and blood (1.86x10^6^ vs 1.85x10^6^) (Fig 8E). Moreover, examination of tissue to blood SPN CFU ratios highlight significantly higher trafficking capability of WT:Ply-Low strain into CNS (Fig 8F) compared to other mice tissues (Fig 8G & 8H). Further, we did not observe any leukocyte infiltration and there were no difference between inflammatory responses in the brains of mice infected with these two SPN strains (Fig 8I & 8J) though SPN was detected in the brain tissue by Gram staining of the brain sections (S7F Fig). This suggests significantly higher transcytosis potential of WT:Ply-Low SPN compared to WT:Ply-High SPN strain into the CNS across an intact BBB. Collectively, we infer that dominance of low Ply expressing subset within heterogeneous SPN population in the blood not only promotes safe passage of bacteria across BBB but also contributes to pneumococcal CNS pathogenesis leading to early death of the host.

### Heterogeneous Ply expression in different SPN serotypes

**Fig 9:**
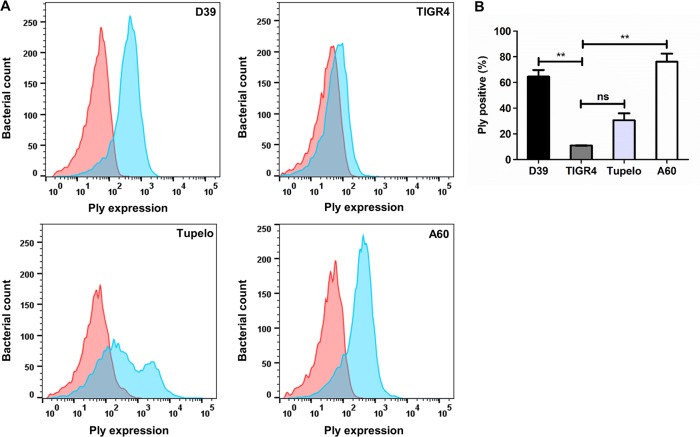
Heterogeneous Ply expression is a serotype independent phenomenon. **A.** Expression of Ply on pneumococcal cell surface was assessed by flow cytometry following staining with Anti-Ply Ab. Different SPN strains used for the study included; D39 (serotype 2), TIGR4 (serotype 4), Tupelo (serotype 14) and A60 (serotype 19F). Pink curve depicts unstained SPN cells while blue curve represents antibody stained SPN cells. Experiments were repeated thrice and representative histograms are shown. **B.** Percentage of Ply positive cells in different SPN serotype strains as analyzed by flow cytometry. Data is represented as mean ± SD of triplicate experiments. Statistical analysis was performed using one-way ANOVA (Tukey’s Multiple comparison test). ns, non-significant; **p<0.005.

Finally, we investigated the phenomenon of Ply heterogeneity in various clinical strains of SPN including D39 (serotype 2), TIGR4 (serotype 4), Tupelo (serotype 14) and A60 (serotype 19F). Interestingly, our flow cytometry analysis for Ply surface expression suggests strain specific differences in Ply levels. Our results demonstrate that irrespective of serotypes, Ply expression is heterogeneous in nature (Fig 9A). Close scrutiny of the flow cytometry data also reflects that highly invasive SPN strains belonging to serotype 4 and 14 contain extremely low number of Ply producers (TIGR4; 10.85% and Tupelo; 30.55%). While the sepsis causing serotype 2 strain D39 (64.5%) and 19F strain (A60; 76.15%) which primarily colonizes human nasopharynx without causing invasive pneumococcal diseases (IPD) exhibited significantly higher Ply expressing population (Fig 9B). This suggests differences in pneumolysin expressing bacterial counts across various SPN serotypes.

## Discussion

A critical trait of any pathogen is its capacity to translocate from the external environment into the host by breaching multiple barriers and reach protected environment such as the CNS for uninhibited proliferation [26]. Most bacterial infections of the CNS are restricted to the meninges and result from dissemination of the pathogens present in the bloodstream. Blood-borne bacteria that invade the CNS should not only have neurotropic attributes, but must also have developed specific mechanisms to circumvent host barriers, particularly BBB. In this study, we for the first time, portrayed a comprehensive picture of intracellular lifestyle of the meningeal pathogen SPN, and its fate during trafficking through BBB while focusing on the role of its major virulence factor, Ply. We first show that abrogation of Ply expression confers a significant survival and trafficking advantage to SPN across the BBB, both *in vitro* and *in vivo*. However, it is noteworthy that a minor subset of WT SPN still survives for longer period which restricts us from declaring Ply to be detrimental for intracellular survival of SPN. Existence of such a dubious role of Ply in pneumococcal intracellular survival propelled us to speculate that cell-to-cell heterogeneity in Ply expression of individual bacterium within the isogenic SPN population could possibly be responsible for the observed disparate infection outcomes. Our studies demonstrate that indeed heterogeneity in Ply expression gives rise to stochastic SPN subsets with variable fate and trafficking efficiency through BBB.

To unravel the role of pneumolysin in the enhanced intracellular killing of SPN, we explored for the contribution of its most important attribute i.e. its pore-forming ability. Endosomal membrane damage induced by pathogen has been demonstrated to trigger anti-bacterial autophagy via recruitment of danger signal molecule, galectin8 [22]. The resultant induction of autophagy, an evolutionary conserved mechanism targeted to remove pathogens following cellular invasion [27–29] involves sequestration of the pathogen in the characteristic LC3-positive double-membrane compartment that finally fuses with lysosomes triggering pathogen clearance. We demonstrated that autophagic targeting of SPN via galectin-8 is strictly dependent on Ply expression and this significantly contributes to pneumococcal clearance inside the brain endothelium. However, despite the robust Ply-driven autophagic targeting of SPN, most of Gal8 positive PCVs, showing association with LC3 and LAMP1, failed to accumulate LysoTracker till delayed hours post infection. It appears therefore that SPN persists inside non-degradative autophagosomal compartments for prolonged period, an assumption, which is additionally supported by our time-lapse analysis for Gal8-SPN association exhibiting slow degradation kinetics of SPN inside autophagosomes. On the basis of these observations we propose the existence of variable maturation kinetics of SPN containing autophagosomes, wherein few show rapid acidification and early bacterial degradation while others prevent acidification and resist their fusion with lysosome contributing to extended survival of a SPN subset inside brain endothelium.

Numerous bacteria escape autophagy via the action of bacterial effector proteins that disrupt vacuolar membrane integrity promoting cytosolic invasion of pathogens [30,31]. Although SPN was shown to reside in the cytoplasm of microglial and epithelial cells, free of any limiting membrane [32,33], the molecular mechanism underlying its eventful progression from the phagosome into cytosol of host cells remained unknown. Our TEM analysis displaying presence of WT SPN in ruptured endosomes and as free cytosolic bacteria, coupled with time-lapse imaging demonstrating pneumococcal escape from autophagosomes clearly implies that Ply mediated extensive damage leads to egress of SPN into cytosol. The cytosolic ubiquitination machinery is known to act as a cellular immune surveillance system promoting clearance of pathogens residing either inside damaged vacuoles or free in cytosol [34]. Although ubiquitination has been reported to result in degradation of SPN in host cells [35,36], our findings showcased that ubiquitination of SPN also occurs strictly in a Ply-dependent manner. Interestingly, only few ubiquitin positive cytosolic SPN get cleared via autophagy pathway while a majority of them degrade in the cytosol in an autophagy-independent manner, most probably via a proteasome dependent mechanism.

**Fig 10:**
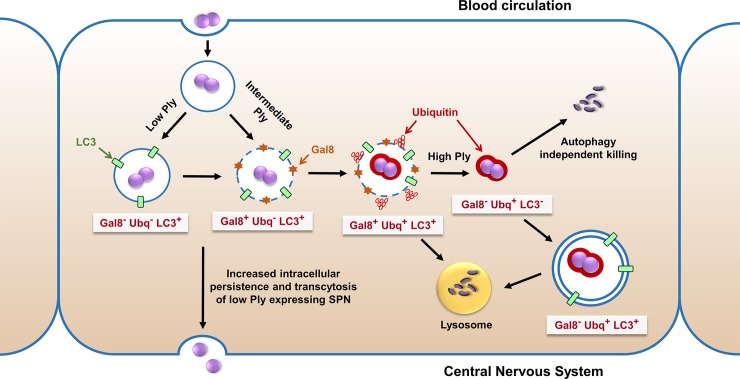
Model depicting variable intracellular fates of SPN due to heterogeneous Ply expression during transit through Blood-brain barrier. Following internalization in brain endothelium SPN starts secreting Ply to halt the vacuole maturation process. However, moderate level of Ply secretion by a subset of vacuolated SPN disrupts endosomal integrity, triggering recruitment of Gal8 and ubiquitin and targeting the SPN towards autophagic clearance. Higher secretion of Ply by a population of SPN further leads to greater damage to the endosomes followed by collapse of the vacuolated structure enabling escape of SPN into cytosol. This cytosolic SPN population is further cleared by ubiquitin mediated autophagy dependent or independent pathways. A small subset of internalized SPN, capable of secreting very low amount of Ply triggers LC3 lipidation of endosomal membrane enabling its longer persistence. This low Ply expressing population preferentially survives and transcytoses across brain endothelium to reach the CNS.

Collectively, our findings for the first time illustrated spatio-temporal heterogeneity in SPN population during trafficking through BBB. While majority of pneumococci undergo early degradation, a few remain unharmed and survive for longer. Some undergo autophagic degradation while others escape autophagy and translocate into the cytosol. Co-localization studies for SPN with the three host markers, Gal8, Ubq and LC3, whose association is strictly Ply-dependent, further substantiates this intracellular heterogeneity and demonstrates the simultaneous existence of different SPN sub-populations which could be correlated to differential expression of Ply within isogenic SPN population. Out of all the subpopulations, the unexpected emergence of the Gal8^-^Ubq^-^LC3^+^ subset was intriguing as it represented Ply dependent LC3 lipidation on PCVs that did not undergo sufficient membrane disruption to recruit the damage sensing markers, Gal8 and Ubq. Osmotic imbalances induced by *H*. *pylori* toxin, VacA has been known to cause such unconventional LC3 lipidation on intact phagosomes via a non-canonical autophagy pathway [37]. Since Ply has been shown to form variable-sized pores on biological membranes and this differential pore-forming ability is directly dependent on the monomer concentration of the toxin [38–40], we surmise that extremely small ion-channel like pores formed by Ply may cause osmotic imbalances triggering LC3 lipidation on a subset of PCVs (represented by Gal8^**–**^ Ubq^**−**^LC3^+^ subset). Indeed the number of Gal8^**–**^ Ubq^**−**^LC3^+^ subset was significantly higher in case of WT:Ply-Low SPN infection that also displayed improved intracellular survival as well as CNS transcytosis capability. This is in accordance with a recent finding that suggests membrane damage leads to exocytosis of bacteria containing vacuole from the host cell in an autophagy dependent manner [41]. On the contrary, in case of WT:Ply-High SPN infection, that comparatively showed lower intracellular survival, the number of Gal8^**–**^ Ubq^+^ LC3^**–**^ subset was significantly higher. This suggested that higher Ply expression triggers irreversible membrane damage leading to collapse of the autophagosomes and escape of SPN into cytosol from where it gets cleared by cytosolic ubiquitination machinery. Overall, these findings showcase a comprehensive picture of the stochastic pneumococcal population subsets interacting with different microbicidal defense pathways as a consequence of heterogeneous expression of pneumolysin inside the brain endothelium (Fig 10).

Different serotypes of SPN have varying potential to cause invasive pneumococcal diseases and are majorly classified into either carriage (15B/C 19F, 23, 35F, etc.) or invasive (1, 4, 5, 7F, 14, etc.) serotypes [42,43]. For years now, although the role of Ply in invasive pneumococcal diseases (IPD) has been widely explored [14,15], its contribution to IPD still remains to be fully elucidated. While studies have shown its crucial role at different stages of disease progression ranging from nasal colonization to invasive diseases like pneumonia, bacteremia and meningitis [15,44,45], discrepancies in the observations by various groups indicate controversial role of this toxin to pneumococcal pathogenesis [10,46]. Our study for the first time elucidated the functional role of heterogeneous Ply expression during pneumococcal BBB trafficking, a crucial step bridging the two important pathological conditions, bacteremia and meningitis. In addition to displaying prolonged intracellular survival, our results showcased higher transcytosis potential of low Ply producing SPN strain into the CNS in an *in vivo* mouse model for bacteremia derived pre-meningitis like condition. These findings were further substantiated by our pneumolysin expression studies across various SPN clinical strains, wherein the widely studied meningitis strain, TIGR4 belonging to the highly invasive serotype 4 [47,48] exhibited extremely low Ply expressing population as compared to the sepsis strain D39 (serotype 2) and the carriage strain A60 (serotype 19F). As high grade bacteremia is prerequisite for meningitis [49], low Ply expression by serotype 4 strains not only ensures threshold bacteremic levels avoiding septic shock and early death of the host which is majorly seen in case of highly proliferating SPN D39 infections [50], but also facilitates safe trafficking across BBB by escaping cellular defense pathways leading to CNS invasion and meningitis as demonstrated by us. Similarly, strains belonging to serotype 14 such as Tupelo, which are most prevalent etiologic agents of pneumococcal community acquired pneumonia (CAP) [51], has Ply expressing population higher than TIGR4 but lower than D39 and A60 strains. Apparently, extremely low Ply levels are required for full virulence of SPN as a mutant strain (Ply^W433F^) exhibiting 0.1% hemolytic activity displayed near-maximum virulence when compared to WT SPN [52]. We further showed that SPN strains harboring such Ply variant retains its ability to disrupt PCV membrane, indicating differences in molecular mechanisms for pore-formation on vacuolar and red blood cell (RBC) membrane. On similar lines, it would be interesting to investigate the molecular mechanisms promoting the high invasive character of non-hemolytic serotype 1 SPN strain ST306 which is a globally disseminated strain causing various IPDs [53].

Virulence factors play both beneficial and detrimental roles during disease progression suggesting that the expression of these factors is finely tuned depending on local environment, promoting diversification of bacterial populations for efficient dissemination inside the host [54]. There is accruing evidence for the existence of such cell-to-cell variation in the expression of numerous bacterial factors [1,55,56] that impacts overall pathogen fitness and subsistence during its challenging journey inside the host. Lately, the bacterial quorum-sensing (QS) systems have been shown to contribute to the phenotypic heterogeneity of genes under their regulation that may result from the dual existence of QS-responsive and QS-non responsive sub-populations within the QS-activated isogenic population [12,57]. It has been suggested that heterogeneity in QS activation or the expression of QS responsive genes may serve as a bet-hedging survival strategy for pathogens which ensures atleast subsets of the heterogeneous population adopt phenotypes which promotes optimal pathogen fitness in the fluctuating environments encountered during disease progression inside the host [43,58]. Pneumolysin expression is reported to be regulated by one such QS system controlled by LuxS that has been shown to play crucial role in pneumococcal virulence and nasal colonization using mouse models of infection [51]. We speculate that this may be responsible for heterogeneous expression of Ply and subsequent genesis of stochastic pneumococcal subpopulations.

It is evident from our studies as well as various earlier reports that success at different stages of pneumococcal pathogenesis are best promoted by varying levels of pneumolysin expression. Metabolically demanding early stages of biofilm formation during SPN colonization in nasopharynx [49] and heart [50] require high Ply expression, conversely low Ply activity imparts early growth advantage to SPN in blood [59] and also promotes prolonged survival and efficient trafficking across BBB, as shown by us. Thus owing to the complex role of Ply at various stages of pneumococcal pathogenesis, generation of phenotypic heterogeneous subsets seems to be a smart strategy adopted by SPN for deploying its most functionally versatile and crucial virulence attribute at various locations inside the host in an economical and proficient manner. We presume that adoption of a Ply-deficient strain to answer the role of Ply in SPN pathogenesis in earlier studies might not have served as an appropriate model to explain the complex and multifactorial role of this important toxin [10,46]. Indeed, our observation of heterogeneity in Ply expression accounts for these conflicting observations for contribution of Ply to pneumococcal pathogenesis.

Having identified the contribution of heterogeneous Ply expression to one aspect of pneumococcal pathogenesis, BBB trafficking, it would be interesting to explore its role to its other facets like nasal colonization, pneumonia and bacteremia. On the basis of our findings and existing literature, we speculate that SPN strains best capable of regulating their Ply stochasticity invade deeper tissues inside the host and cause wide range of IPDs. Thus understanding the role and mechanism behind stochastic Ply expression in the different SPN strains (carriage and invasive) may not only provide clues for decoding the perennial mystery of clonal evolution that fosters global dissemination of pathogens but is also pivotal for development of novel treatment strategies to combat the diverse and lethal pneumococcal diseases.

## Supporting information

S1 Fig Effect of Ply on intracellular persistence of SPN in brain endothelium is independent of serotype and capsule. **A & C.** Percent hemolysis of wildtype (WT), *ply* mutant (Δ*ply*) and complemented (Δ*ply*:pPly) strains of SPN strain R6 (serotype 2) **(A)** or wild type (WT), Δ*ply*, Δ*cps* (capsule mutant) and Δ*cps*Δ*ply* (capsule-pneumolysin double mutant) of SPN strain TIGR4 (serotype 4) **(C)** relative to positive control (0.05% Triton X-100). Data are presented as mean ± SD of triplicate experiments. Statistical analysis was performed using one-way ANOVA (Tukey’s multiple comparison test). ns, nonsignificant; **p<0.005; ***p<0.001.
**B.** Viability of hBMECs following infection with WT and Δ*ply* mutant SPN strains as determined by MTT assay. Uninfected cells were used as a negative control. Results are expressed as percent cell viability with respect to negative control. Bars are mean ± SD. Statistical analysis was performed using one-way ANOVA (Tukey’s multiple comparison test); ns, non-significant; **p < 0.005.
**D & E.** Intracellular survival efficiency of WT encapsulated serotype 4 strain TIGR4 and its Δ*ply* mutant (**D**) and Δ*cps* and Δ*cps*Δ*ply* strains (**E**) in hBMECs were calculated as percent survival at indicated time points relative to 0 h. Data are presented as mean ± SD of triplicate experiments. Statistical analysis was performed using two-way ANOVA (Bonferroni test). ns, non-significant; *p<0.05; **p<0.005.
**F**. Percent co-localization of Gal8 with WT TIGR4 and its Δ*ply* derivative at 2 h post-infection. n ≥ 100 bacteria per coverslip. Data are presented as mean ± SD of triplicate hBMEC cultures.
(TIF)

S2 Fig Lack of Ply expression promotes pneumococcal trafficking across BBB. Balb/c mice were infected i.v. with 10^6^ CFU of SPN TIGR4 WT or Δ*ply* strain and were sacrificed at 14 h p.i. PBS injected mice served as uninfected control.
**A.** Quantification of bacterial counts (CFU) in various tissue homogenates and blood of mice infected with different SPN strains. Each dot represents one mouse; black bars show average values. n = 5 per group. Statistical analysis for each tissue was individually performed using non-parametric test (Mann-Whitney test). *p* values are mentioned in the graph.
**B—D.** Ratio of bacterial CFU in brain to blood **(B)**, lungs to blood **(C)** and spleen to blood **(D)** of individual infected mice. Each dot represents one mouse; black bars show average values. n = 5 per group. Statistical analysis was performed using non-parametric test (Mann-Whitney test). *p* values are mentioned in the graph.
**E.** Transcript abundance of pro-inflammatory cytokines in total RNA isolated from brains of mice that were injected with PBS or infected with different SPN strains. Transcript levels were normalized to 18S rRNA and expressed as fold change compared to control mice. Statistical analysis was performed using two-way ANOVA (Bonferroni test); ns, non-significant.
**F.** Histopathology of H & E stained representative brain tissue samples of control (PBS injected) or mice infected with different strains of SPN. Scale bar, 100 μm.
(TIF)

S3 Fig Non-hemolytic Ply^W433F^ variant ruptures endosomes and targets SPN towards autophagy. **A.** Western blot demonstrating similar level of Ply expression in WT and WT:Ply^W433F^ strain. Enolase served as loading control.
**B**. Hemolytic activity of WT and WT:Ply^W433F^ strain relative to positive control (0.05% Triton X-100). Data are presented as mean ± SD of triplicate experiments. Statistical analysis was performed using one-way ANOVA (Tukey’s multiple comparison test). ***p<0.001.
**C**. Association of WT and WT:Ply^W433F^ SPN strains with Gal8 at 6 h post-infection. n ≥ 100 bacteria per coverslip. Data are presented as mean ± SD of triplicate hBMEC cultures.
**D**. Comparison of intracellular survival efficiencies between WT and WT:Ply^W433F^ strains in hBMECs expressed as percent survival at indicated time points relative to 0 h. Data are presented as mean ± SD of triplicate experiments. Statistical analysis was performed using two-way ANOVA (Bonferroni test); ns: non-significant.
(TIF)

S4 Fig Contribution of autophagy for clearance of intracellular SPN in brain endothelium. **A.** Confocal micrographs showing association of LC3-GFP (green) with tRFP expressing SPN (red) inside hBMECs. Arrowhead designates bacteria shown in insets. Events are localized at z-stack no. 3 (out of 7) and 6 (out of 18) for WT and Δ*ply*, respectively. Scale bar, 5 μm.
**B.** Quantification of association of LC3 with WT and Δ*ply* SPN strains at indicated time points p.i. n ≥ 100 bacteria per coverslip. Data are presented as mean ± SD of triplicate hBMEC cultures.
**C.** Intracellular survival percentages of WT SPN following treatment with 3-Methyl Adenine (1 mM) at indicated time points relative to 0 h. Data are presented as mean ± SD of triplicate experiments. Statistical analysis was performed using two-way ANOVA (Bonferroni test). ns, non-significant, *p<0.05, **p<0.005.
**D.** Western blot demonstrating LC3 flux in hBMECs following infection with WT and Δ*ply* strains at indicated time points p.i. in presence or absence of Bafilomycin A1 (100 nM). Untreated cells (U) served as negative control. Rapamycin (2.5 μM) was used as positive control for autophagy induction. GAPDH served as house-keeping gene.
(TIF)

S5 Fig Autophagic targeting of SPN is Ply dependent. **A.** Intracellular survival of Δ*ply* mutant strain in hBMECs following transfection with siGal8 or siControl at indicated time points relative to 0 h. Data are presented as mean ± SD of triplicate hBMEC cultures. Statistical analysis was performed using two-way ANOVA (Bonferroni test). ns, non-significant.
**B.** Quantification of LAMP1 positive intracellular bacteria at 1 h following infection of hBMECs with WT SPN and Δ*ply* mutant strains. n ≥ 100 bacteria per coverslip. Data are presented as mean ± SD of triplicate hBMEC cultures. Statistical analysis was performed using Students t-test. **p<0.005.
**C—D.** Transmission electron micrograph depicting Δ*ply* mutant **(C)** in intact membrane bound vacuolar compartments inside hBMECs. Scale bar, 2 μm. Zoomed in view of the boxed area in “**C**” is shown in “**D**”. Scale bar, 0.5 μm.
**E.** Intracellular survival of Δ*ply* mutant strain in hBMECs following pretreatment with E1 ubiquitin activating enzyme inhibitor PYR41 (45 μM) at indicated time points relative to 0 h. Data are presented as mean ± SD of triplicate hBMEC cultures. Statistical analysis was performed using two-way ANOVA (Bonferroni test). ns, non-significant.
(TIF)

S6 Fig Heterogeneous expression of Ply in SPN population. **A.** Quantification of different population subsets of intracellular WT SPN at 6 h p.i. for association with Gal8, Ubq and LC3. n ≥ 100 bacteria per coverslip. Data are presented as mean ± SD of triplicate experiments.
**B.** Percent hemolysis of WT and WT::Ply:GFP transcriptional fusion strain relative to positive control (0.05% Triton X-100). Data are presented as mean ± SD of triplicate experiments. Statistical analysis was performed using one-way ANOVA (Tukey’s multiple comparison test). ns, non-significant.
**C.** Flow cytometry analysis of Ply surface expression in WT::Ply:GFP strain. Pink curve depicts unstained SPN cells while blue curve represents antibody stained SPN cells. Experiments were repeated thrice and representative histograms are shown.
(TIF)

S7 Fig Differential Ply expression in various SPN strains. **A.** Western blot demonstrating differential Ply expression in WT:Ply-High and WT:Ply-Low SPN strains compared to WT SPN (in both R6 and TIGR4 background). Enolase served as loading control.
**B.** Densitometric analysis of the Ply relative to Enolase bands in three independent western blotting experiments. Statistical analysis was performed using One-way ANOVA (Tukey’s multiple comparison test). *p<0.05; ***p<0.001.
**C.** Flow cytometry analysis of surface Ply expression in differential Ply expressing SPN strains. Pink curve depicts unstained SPN cells while blue curve represents anti-Ply antibody stained SPN cells. Experiments were repeated thrice and representative histograms are shown.
**D.** Percentage of Ply positive cells in differential Ply expressing SPN strains as analyzed by flow cytometry.
**E.** Comparison of invasion rate of WT:Ply-High and WT:Ply-Low SPN strains in hBMECs as assessed by penicillin-gentamycin protection assay. Data are presented as mean ± SD of triplicate experiments. Statistical analysis was performed using Students t-test. ns, nonsignificant.
**F.** Gram staining of mice brain sections following treatment with PBS (Control) or infection with WT:Ply-Low strain for detection of pneumococci inside brain tissue. Scale bar, 20 μm. Zoomed in view of the boxed area in “**ii**” is shown in “**iii**”.
(TIF)

S1 Movie Galectin 8 mediated intravacuolar killing of SPN. Time lapse imaging of tRFP expressing WT SPN (red) infected hBMECs stably expressing YFP-Gal8 (green). Live cell imaging was performed under spinning disc confocal microscope starting at 3 h p.i. The video depicts degradation of SPN while being associated with Gal8 signal. Time interval, 5 min; scale bar, 2 μm.
(AVI)

S2 Movie Vacuolar escape and Galectin 8 independent clearance of SPN. Time lapse imaging of hBMECs stably expressing YFP-Gal8 (green) infected with tRFP expressing WT SPN (red). Live cell imaging was performed under spinning disc confocal microscope starting at 3 h p.i. The video depicts collapse of Gal8 marked compartments followed by escape of SPN in cytosol and eventual degradation. Time interval, 5 min; scale bar, 2 μm.
(AVI)

S3 Movie Ubiquitination and autophagy dependent killing of SPN. Time lapse imaging of degradation of DRAQ5 stained WT SPN (white) inside hBMECs stably expressing GFP-LC3 (green) and Ubq-mStrawberry (red). Live cell imaging was performed under laser scanning confocal microscope starting at 6 h p.i. The video depicts degradation of ubiquitinated cytosolic SPN in an autophagy-dependent manner (LC3 dependent killing). Time interval, 10 min; scale bar, 1.5 μm.
(AVI)

S4 Movie Clearance of ubiquitinated SPN in autophagy independent manner. Time lapse imaging of degradation of DRAQ5 stained WT SPN (white) inside hBMECs stably expressing GFP-LC3 (green) and Ubq-mStrawberry (red). Live cell imaging was performed under laser scanning confocal microscope starting at 6 h p.i. The video depicts autophagy-independent degradation of ubiquitinated cytosolic SPN (LC3 independent killing). Time interval, 10 min; scale bar, 1.5 μm.
(AVI)

S1 Table Sequences of oligonucleotides. (DOCX)
