# Supplementary figures and images for: Heterogeneity in pneumolysin expression governs the fate of Streptococcus pneumoniae during blood-brain barrier trafficking
Source: PLoS Pathog. 2018 Jul 16;14(7):e1007168. doi: 10.1371/journal.ppat.1007168 (PMC6062133; doi:10.1371/journal.ppat.1007168)

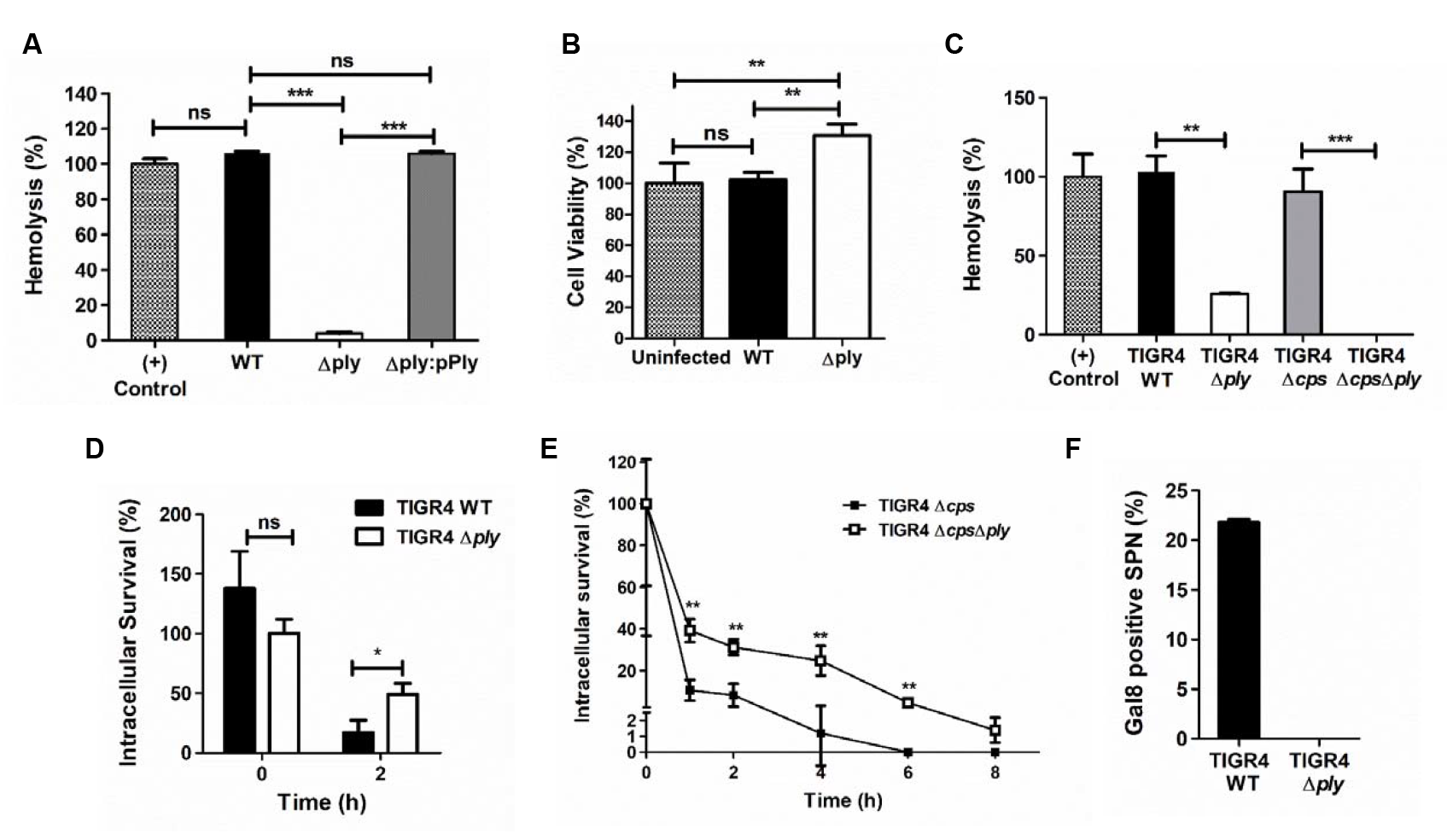

Supplement: S1 Fig — A & C. Percent hemolysis of wildtype (WT), ply mutant (Δply) and complemented (Δply:pPly) strains of SPN strain R6 (serotype 2) (A) or wild type (WT), Δply, Δcps (capsule mutant) and ΔcpsΔply (capsule-pneumolysin double mutant) of SPN strain TIGR4 (serotype 4) (C) relative to positive control (0.05% Triton X-100). Data are presented as mean ± SD of triplicate experiments. Statistical analysis was performed using one-way ANOVA (Tukey’s multiple comparison test). ns, nonsignificant; **p<0.005; ***p<0.001. B. Viability of hBMECs following infection with WT and Δply mutant SPN strains as determined by MTT assay. Uninfected cells were used as a negative control. Results are expressed as percent cell viability with respect to negative control. Bars are mean ± SD. Statistical analysis was performed using one-way ANOVA (Tukey’s multiple comparison test); ns, non-significant; **p < 0.005. D & E. Intracellular survival efficiency of WT encapsulated serotype 4 strain TIGR4 and its Δply mutant (D) and Δcps and ΔcpsΔply strains (E) in hBMECs were calculated as percent survival at indicated time points relative to 0 h. Data are presented as mean ± SD of triplicate experiments. Statistical analysis was performed using two-way ANOVA (Bonferroni test). ns, non-significant; *p<0.05; **p<0.005. F. Percent co-localization of Gal8 with WT TIGR4 and its Δply derivative at 2 h post-infection. n ≥ 100 bacteria per coverslip. Data are presented as mean ± SD of triplicate hBMEC cultures. (TIF) [file ppat.1007168.s001.tif]

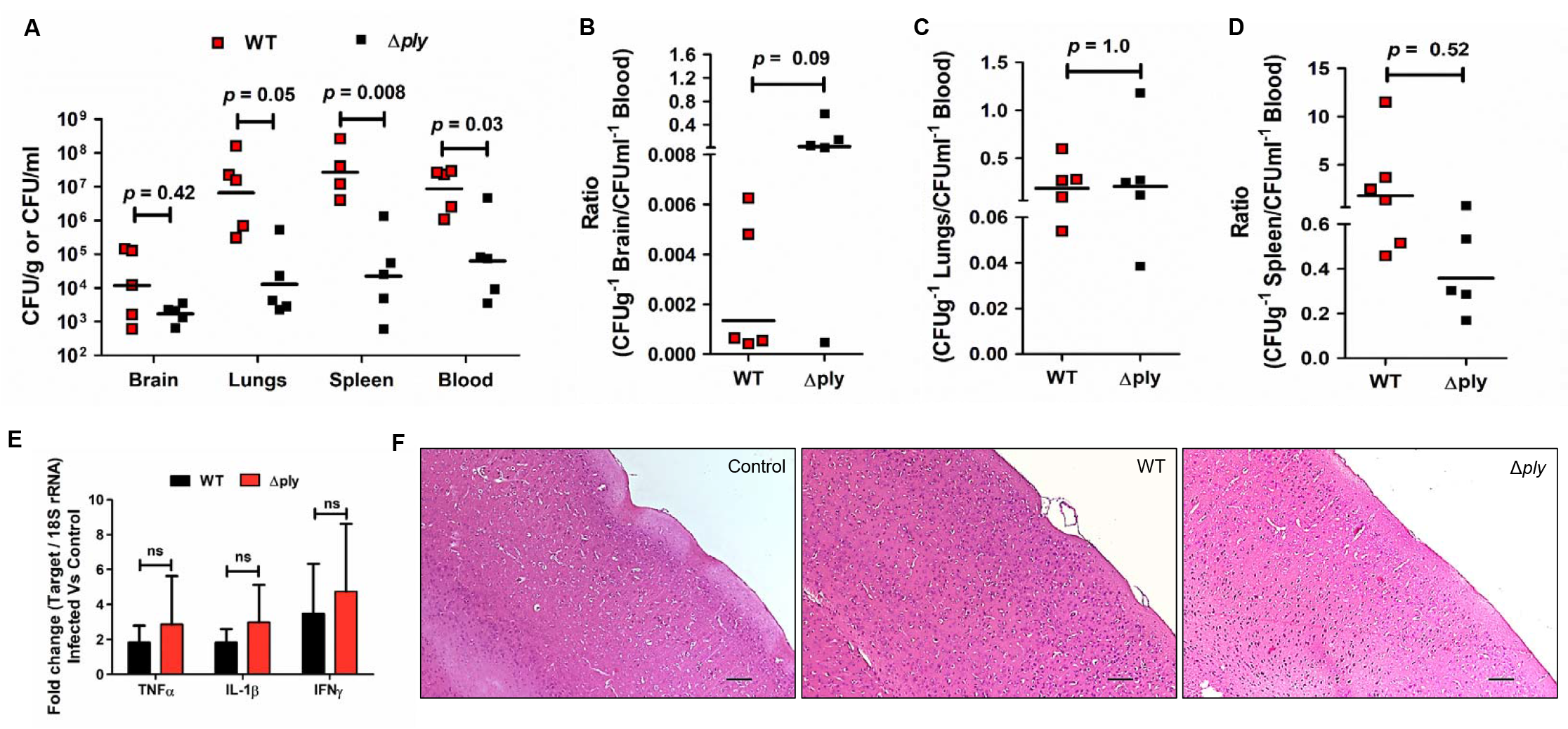

Supplement: S2 Fig — Balb/c mice were infected i.v. with 106 CFU of SPN TIGR4 WT or Δply strain and were sacrificed at 14 h p.i. PBS injected mice served as uninfected control. A. Quantification of bacterial counts (CFU) in various tissue homogenates and blood of mice infected with different SPN strains. Each dot represents one mouse; black bars show average values. n = 5 per group. Statistical analysis for each tissue was individually performed using non-parametric test (Mann-Whitney test). p values are mentioned in the graph. B—D. Ratio of bacterial CFU in brain to blood (B), lungs to blood (C) and spleen to blood (D) of individual infected mice. Each dot represents one mouse; black bars show average values. n = 5 per group. Statistical analysis was performed using non-parametric test (Mann-Whitney test). p values are mentioned in the graph. E. Transcript abundance of pro-inflammatory cytokines in total RNA isolated from brains of mice that were injected with PBS or infected with different SPN strains. Transcript levels were normalized to 18S rRNA and expressed as fold change compared to control mice. Statistical analysis was performed using two-way ANOVA (Bonferroni test); ns, non-significant. F. Histopathology of H & E stained representative brain tissue samples of control (PBS injected) or mice infected with different strains of SPN. Scale bar, 100 μm. (TIF) [file ppat.1007168.s002.tif]

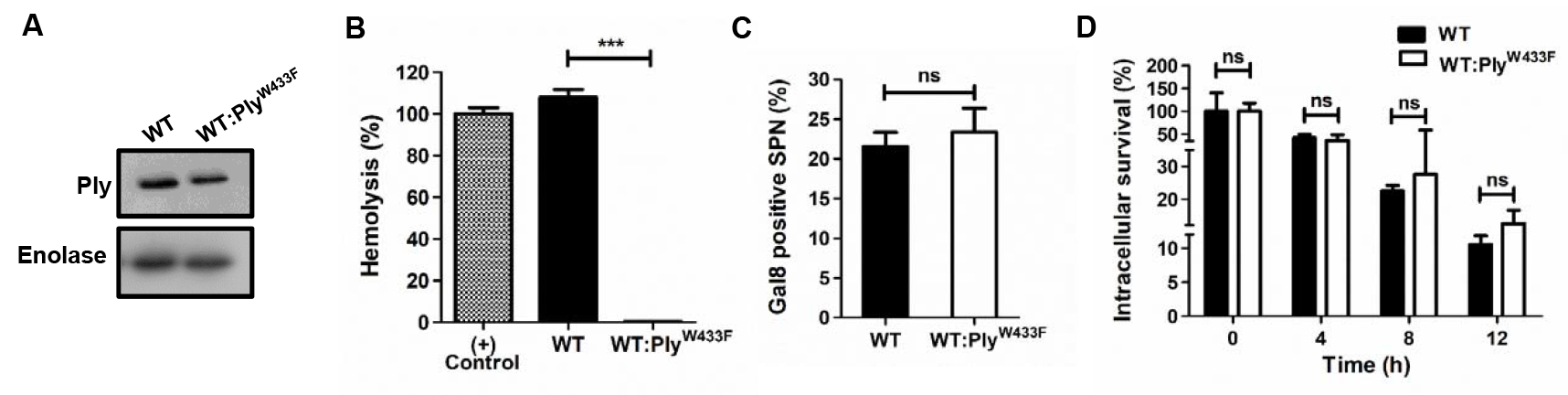

Supplement: S3 Fig — A. Western blot demonstrating similar level of Ply expression in WT and WT:PlyW433F strain. Enolase served as loading control. B. Hemolytic activity of WT and WT:PlyW433F strain relative to positive control (0.05% Triton X-100). Data are presented as mean ± SD of triplicate experiments. Statistical analysis was performed using one-way ANOVA (Tukey’s multiple comparison test). ***p<0.001. C. Association of WT and WT:PlyW433F SPN strains with Gal8 at 6 h post-infection. n ≥ 100 bacteria per coverslip. Data are presented as mean ± SD of triplicate hBMEC cultures. D. Comparison of intracellular survival efficiencies between WT and WT:PlyW433F strains in hBMECs expressed as percent survival at indicated time points relative to 0 h. Data are presented as mean ± SD of triplicate experiments. Statistical analysis was performed using two-way ANOVA (Bonferroni test); ns: non-significant. (TIF) [file ppat.1007168.s003.tif]

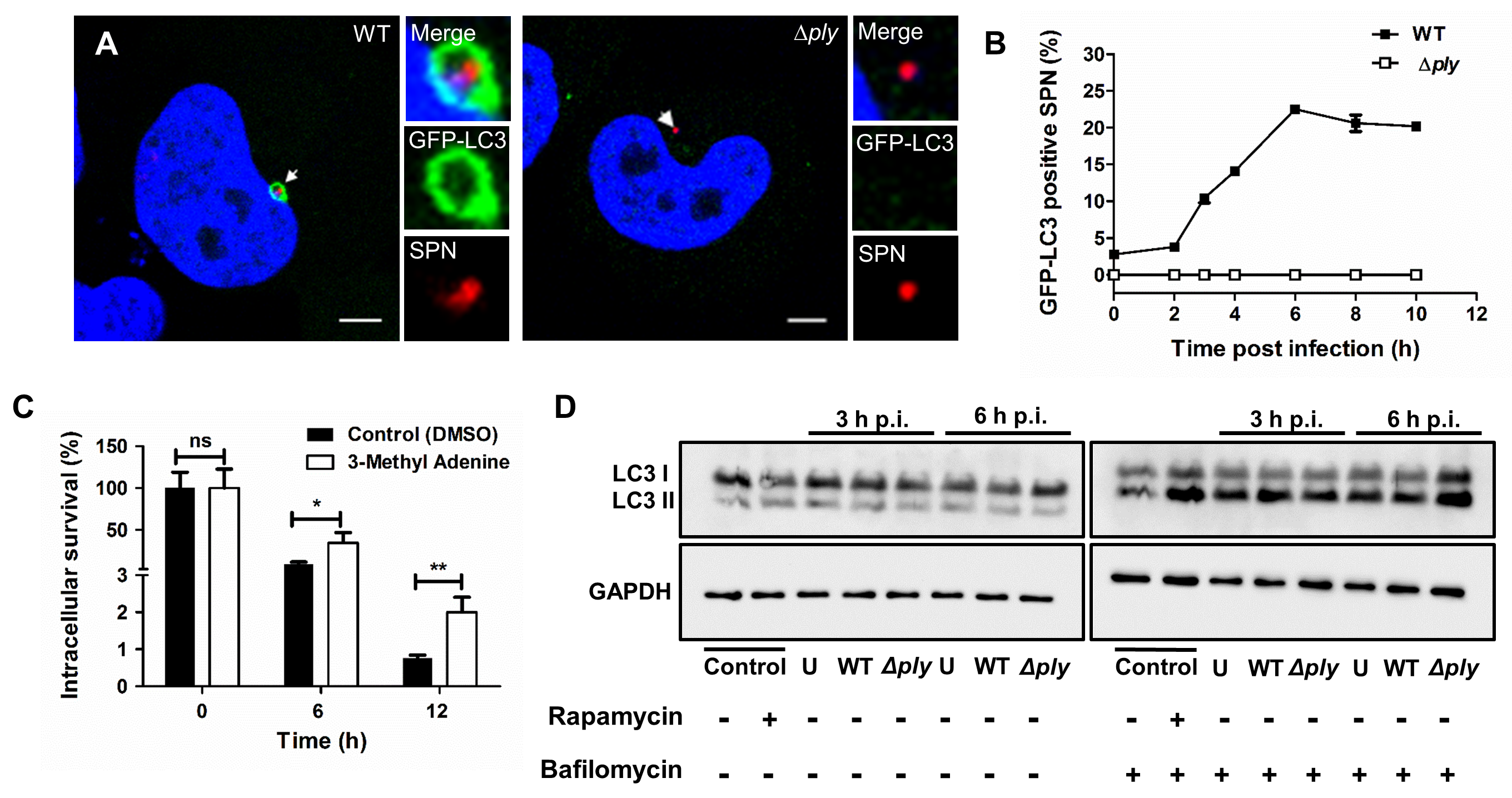

Supplement: S4 Fig — A. Confocal micrographs showing association of LC3-GFP (green) with tRFP expressing SPN (red) inside hBMECs. Arrowhead designates bacteria shown in insets. Events are localized at z-stack no. 3 (out of 7) and 6 (out of 18) for WT and Δply, respectively. Scale bar, 5 μm. B. Quantification of association of LC3 with WT and Δply SPN strains at indicated time points p.i. n ≥ 100 bacteria per coverslip. Data are presented as mean ± SD of triplicate hBMEC cultures. C. Intracellular survival percentages of WT SPN following treatment with 3-Methyl Adenine (1 mM) at indicated time points relative to 0 h. Data are presented as mean ± SD of triplicate experiments. Statistical analysis was performed using two-way ANOVA (Bonferroni test). ns, non-significant, *p<0.05, **p<0.005. D. Western blot demonstrating LC3 flux in hBMECs following infection with WT and Δply strains at indicated time points p.i. in presence or absence of Bafilomycin A1 (100 nM). Untreated cells (U) served as negative control. Rapamycin (2.5 μM) was used as positive control for autophagy induction. GAPDH served as house-keeping gene. (TIF) [file ppat.1007168.s004.tif]

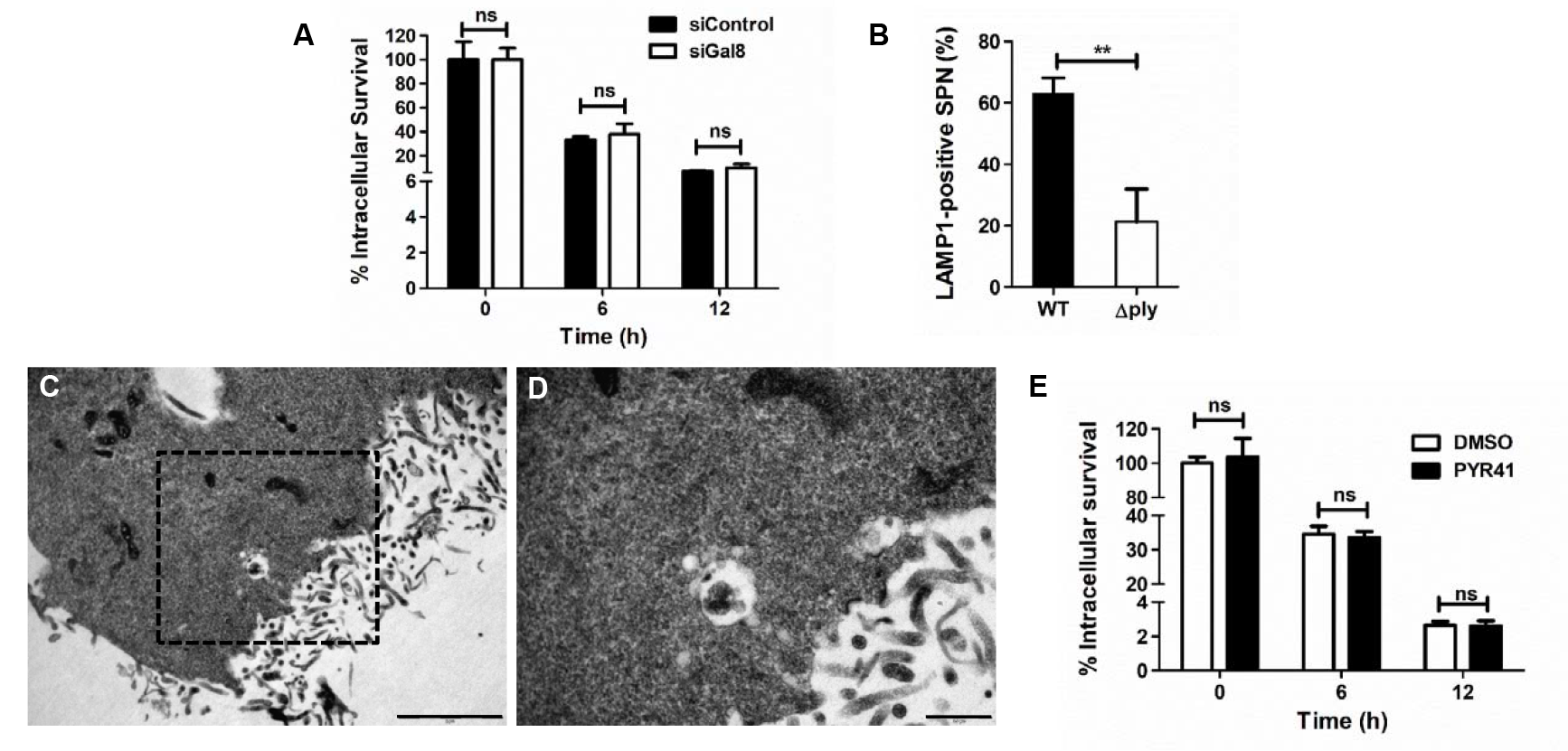

Supplement: S5 Fig — A. Intracellular survival of Δply mutant strain in hBMECs following transfection with siGal8 or siControl at indicated time points relative to 0 h. Data are presented as mean ± SD of triplicate hBMEC cultures. Statistical analysis was performed using two-way ANOVA (Bonferroni test). ns, non-significant. B. Quantification of LAMP1 positive intracellular bacteria at 1 h following infection of hBMECs with WT SPN and Δply mutant strains. n ≥ 100 bacteria per coverslip. Data are presented as mean ± SD of triplicate hBMEC cultures. Statistical analysis was performed using Students t-test. **p<0.005. C—D. Transmission electron micrograph depicting Δply mutant (C) in intact membrane bound vacuolar compartments inside hBMECs. Scale bar, 2 μm. Zoomed in view of the boxed area in “C” is shown in “D”. Scale bar, 0.5 μm. E. Intracellular survival of Δply mutant strain in hBMECs following pretreatment with E1 ubiquitin activating enzyme inhibitor PYR41 (45 μM) at indicated time points relative to 0 h. Data are presented as mean ± SD of triplicate hBMEC cultures. Statistical analysis was performed using two-way ANOVA (Bonferroni test). ns, non-significant. (TIF) [file ppat.1007168.s005.tif]

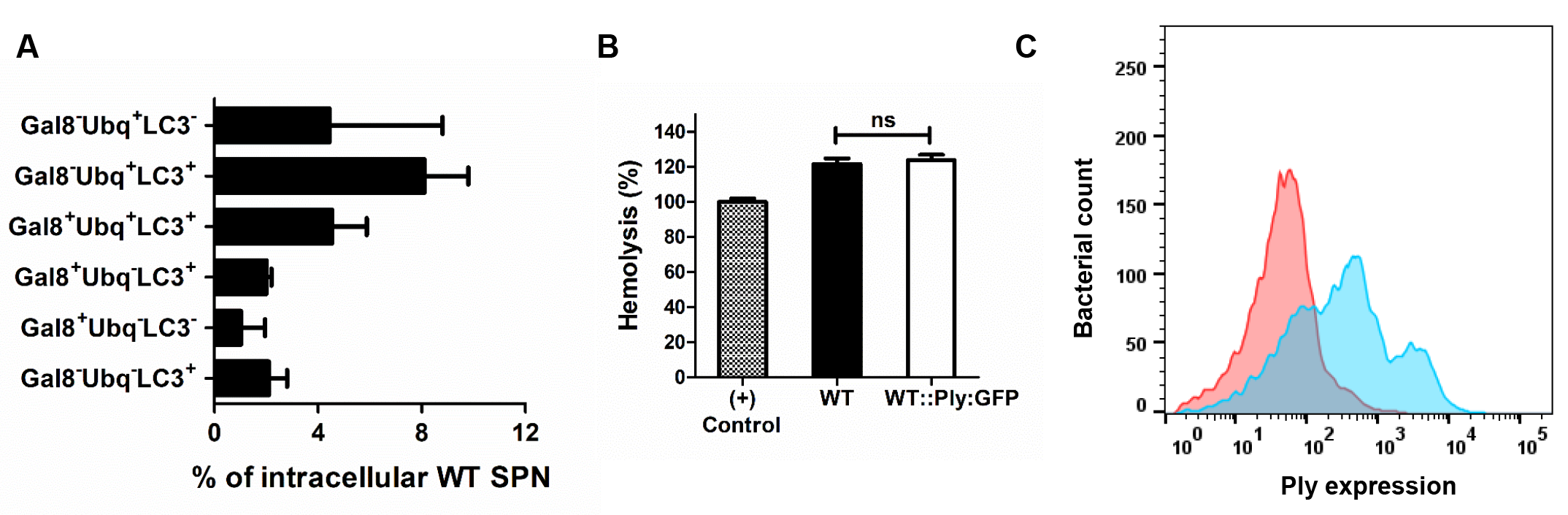

Supplement: S6 Fig — A. Quantification of different population subsets of intracellular WT SPN at 6 h p.i. for association with Gal8, Ubq and LC3. n ≥ 100 bacteria per coverslip. Data are presented as mean ± SD of triplicate experiments. B. Percent hemolysis of WT and WT::Ply:GFP transcriptional fusion strain relative to positive control (0.05% Triton X-100). Data are presented as mean ± SD of triplicate experiments. Statistical analysis was performed using one-way ANOVA (Tukey’s multiple comparison test). ns, non-significant. C. Flow cytometry analysis of Ply surface expression in WT::Ply:GFP strain. Pink curve depicts unstained SPN cells while blue curve represents antibody stained SPN cells. Experiments were repeated thrice and representative histograms are shown. (TIF) [file ppat.1007168.s006.tif]

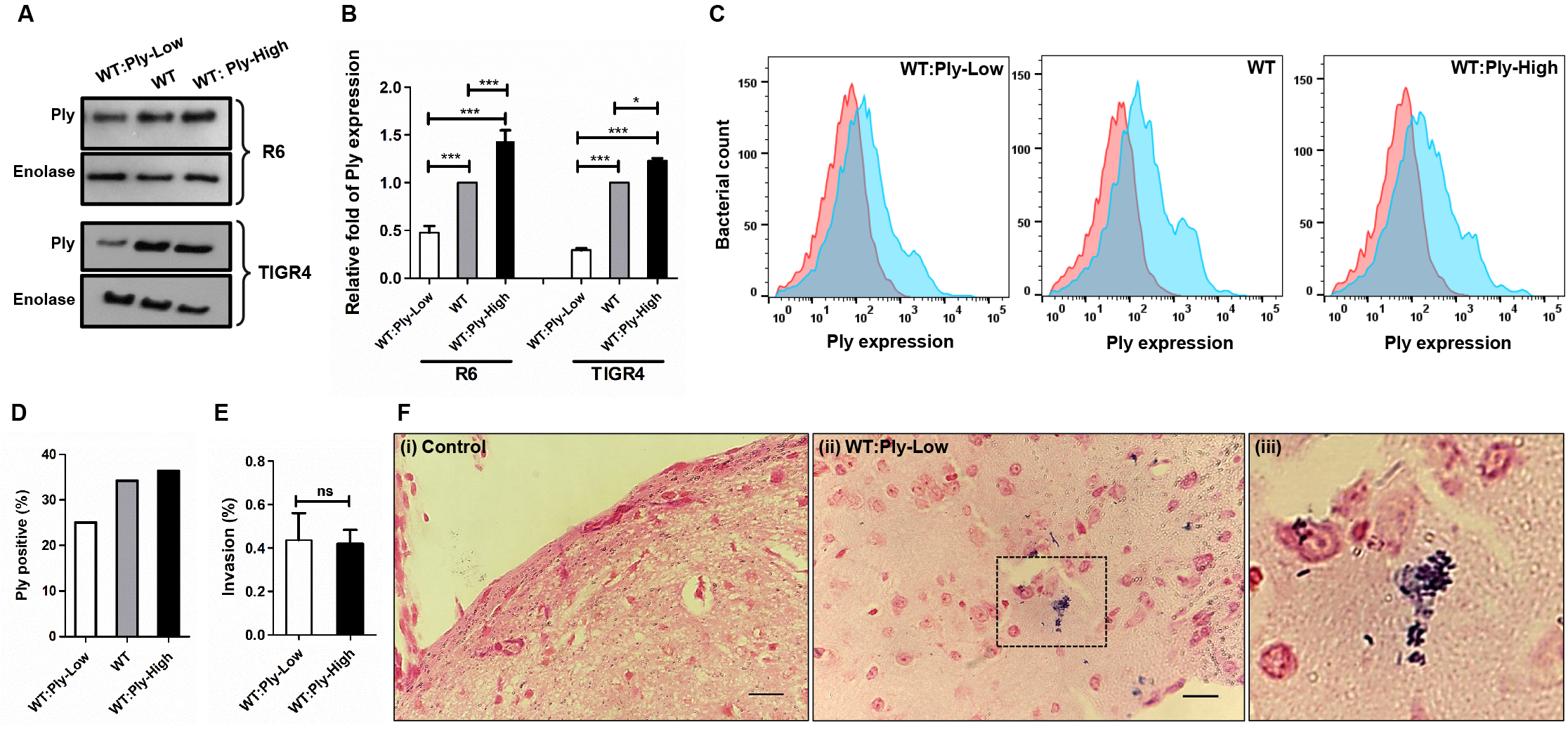

Supplement: S7 Fig — A. Western blot demonstrating differential Ply expression in WT:Ply-High and WT:Ply-Low SPN strains compared to WT SPN (in both R6 and TIGR4 background). Enolase served as loading control. B. Densitometric analysis of the Ply relative to Enolase bands in three independent western blotting experiments. Statistical analysis was performed using One-way ANOVA (Tukey’s multiple comparison test). *p<0.05; ***p<0.001. C. Flow cytometry analysis of surface Ply expression in differential Ply expressing SPN strains. Pink curve depicts unstained SPN cells while blue curve represents anti-Ply antibody stained SPN cells. Experiments were repeated thrice and representative histograms are shown. D. Percentage of Ply positive cells in differential Ply expressing SPN strains as analyzed by flow cytometry. E. Comparison of invasion rate of WT:Ply-High and WT:Ply-Low SPN strains in hBMECs as assessed by penicillin-gentamycin protection assay. Data are presented as mean ± SD of triplicate experiments. Statistical analysis was performed using Students t-test. ns, nonsignificant. F. Gram staining of mice brain sections following treatment with PBS (Control) or infection with WT:Ply-Low strain for detection of pneumococci inside brain tissue. Scale bar, 20 μm. Zoomed in view of the boxed area in “ii” is shown in “iii”. (TIF) [file ppat.1007168.s007.tif]
