# Supplementary material for: Heterogeneity in pneumolysin expression governs the fate of Streptococcus pneumoniae during blood-brain barrier trafficking
Source: PLoS Pathog. 2018 Jul 16;14(7):e1007168. doi: 10.1371/journal.ppat.1007168 (PMC6062133; doi:10.1371/journal.ppat.1007168)
Supplement: S1 Table — (DOCX) [file ppat.1007168.s012.docx]

| **Suppl. Table 1. List of oligonucleotides.** | |
| --- | --- |
|  | |
| **Primer** | **Sequence (5' to 3')** |
| ply-F1 | GTCGCAAGCATTCTCCTCTC |
| ply-R1 | ACTTAGTCCAACCACGGCTG |
| dexB-F-SacI | ATGC**GAGCTC**GAAGATATTGAATCTCTCAACT |
| dexB-R-BamHI | CGGC**GGATCC**TTATAGTAATTCCACACAGA |
| spec-F-BamHI | CCGC**GGATCC**CATATATAATCTAGAATAAAATTAAC |
| spec-R-BamHI | ATCC**GGATCC**AATCTGATTACCAATTAGAATG |
| aliA-F-BamHI | GGCC**GGATCC**ATGATGAAAAGTTCAAAACTAT |
| aliA-R-XhoI | GAGC**CTCGAG**CCTTTTGAATTCAAAAACTCTT |
| ply-F3-BamHI | AAGGA**GGATCC**ATGGCAAATAAAGCAGTAAA |
| ply-R3-SacI | TCCAA**GAGCTC**CTAGTCATTTTCTACCTTAT |
| ply-wt-XhoI-F | TATATA**CTCGAG**TTAGGAGGTAGAAGATGGCA |
| ply-wt-BamHI-R | ATATAT**GGATCC**TCTCCTCTCCTAGTCATTTT |
| ply-sdm1-F | GAAGATGGCTAATAAGGCGGTGAACGACTTTATCCTAGCAATGAATTACG |
| ply-sdm1-R | CGTAATTCATTGCTAGGATAAAGTCGTTCACCGCCTTATTAGCCATCTTC |
| ply-sdm2-F | GAAAATGGCAAACAAGGCTGTTAATGACTTTATACTAGCTATGAATTACG |
| ply-sdm2-R | CGTAATTCATAGCTAGTATAAAGTCATTAACAGCCTTGTTTGCCATTTTC |
| ply-F-W433F | GAGAGTGTACCGGGCTTGCCTTTGAATGGTGGCGTACGGTTTA |
| ply-R-W433F | TAAACCGTACGCCACCATTCAAAGGCAAGCCCGGTACACTCTC |
| ply-up-KpnI-F2 | TCTAAT**GGTACC**GGTTATTGGCGACAAGCATT |
| ply-up-XhoI-R2 | ATCCCC**CTCGAG**TAAGTTCCTGGATT |
| ply-dwn-BamHI-F2 | TATATA**GGATCC**ATGCTTGCGACAAAAAGAGG |
| ply-dwn-XbaI-R | AGTGGA**TCTAGA**CAGTTCTTATAGGCGCTATTGC |
| ply- XhoI-F2 | CATCCT**CTCGAG**ATGGCAAATAAAGCAGTA |
| ply-XmaI -R | ATTATA**CCCGGG**CTAGTCATTTTCTACCTTATC |
| GFP-XmaI-F1 | TAATT**CCCGGG**AGGAGGTAAATCTAATGTCAAAAGGAGAAGAGCTG |
| GFP-BamHI-R | GCTGC**GGATCC**TTACTTATAAAGCTCATCCATGCCGTG |
| IL-1β-F | CTGATGAGAGCATCCAGCTTCA |
| IL-1β-R | CTTCTTTGGGTATTGCTTGGGATC |
| TNFα-F | TCTTCTCATTCCTGCTTGTGG |
| TNFα-R | GGTCTGGGCCATAGAACTGA |
| IFNγ-F | GGCCATCAGCAACAACATAAGCGT |
| IFNγ-R | TGGGTTGTTGACCTCAAACTTGGC |
| 18S rRNA-F | AACCCGGTGAGCTCCCTCCC |
| 18S rRNA-R | TTCGAATGGGTCGTCGCCGC |
| RT-ply-F | TGAGACTAAGGTTACAGCTTACAG |
| RT-ply-R | CTAATTTTGACAGAGAGATTACGA |
| 16S rRNA-F | TGGTTGTCGTCAGCTCGTGT |
| 16S rRNA-R | GGCTTGCGACTCGTTGTACC |
